# A *Citrullus* genus super‐pangenome reveals extensive variations in wild and cultivated watermelons and sheds light on watermelon evolution and domestication

**DOI:** 10.1111/pbi.14120

**Published:** 2023-07-25

**Authors:** Shan Wu, Honghe Sun, Lei Gao, Sandra Branham, Cecilia McGregor, Susanne S. Renner, Yong Xu, Chandrasekar Kousik, W. Patrick Wechter, Amnon Levi, Zhangjun Fei

**Affiliations:** ^1^ Boyce Thompson Institute Ithaca New York USA; ^2^ Plant Biology Section, School of Integrative Plant Science Cornell University Ithaca New York USA; ^3^ CAS Key Laboratory of Plant Germplasm Enhancement and Specialty Agriculture, Wuhan Botanical Garden, Innovative Academy of Seed Design Chinese Academy of Sciences Wuhan Hubei China; ^4^ Coastal Research and Educational Center Clemson University Charleston South Carolina USA; ^5^ Department of Horticulture University of Georgia Athens Georgia USA; ^6^ Department of Biology Washington University Saint Louis Missouri USA; ^7^ National Engineering Research Center for Vegetables Beijing Academy of Agriculture and Forestry Sciences Beijing China; ^8^ USDA‐ARS U.S. Vegetable Laboratory Charleston South Carolina USA; ^9^ USDA‐ARS Robert W. Holley Center for Agriculture and Health Ithaca New York USA

**Keywords:** watermelon, super‐pangenome, wild relatives, domestication, disease resistance

Cultivated watermelon (*Citrullus lanatus* subsp. *vulgaris*) has a narrow genetic base due to domestication and breeding focusing primarily on fruit quality traits. Bitter or bland‐tasting wild watermelons, such as *C. mucosospermus*, *C. amarus* and *C. colocynthis*, have been used in watermelon breeding to introduce disease resistance to modern cultivars (Levi *et al*., 2017). These wild relatives are valuable sources for broadening the improvement potential of cultivated watermelon, providing additional functionally important genes and alleles that are absent in cultivated watermelon. However, the lack of genome sequences of these wild watermelon species has limited their utilization in watermelon breeding.

In this study, we assembled high‐quality reference genomes for three wild watermelons. Three wild accessions were selected for reference genome sequencing: *C. mucosospermus* USVL531‐MDR, *C. amarus* USVL246‐FR2 and *C. colocynthis* PI 537277. PacBio CLR sequences for USVL531‐MDR and Illumina sequences for USVL246‐FR2 and PI 537277 were generated (Table S1; Figure S1) and *de novo* assembled. Each of the resulting assemblies (Table S2) was anchored to 11 chromosomes (Table S3; Figures S2 and S3; Appendix S1). Quality assessments demonstrated high quality of these assemblies (Appendix S1). The repeat‐masked assemblies (Table S4) were annotated for protein‐coding genes. Gene predictions in six watermelon reference genomes, including three developed here and three published ones (Guo *et al*., 2019; Renner *et al*., 2021; Wu *et al*., 2019), were improved through mapping genes between assemblies with Liftoff (Shumate and Salzberg, 2021). A total of 21 676 to 22 764 protein‐coding genes were predicted in these six watermelon genomes (Table S5). Comparative analysis revealed a large inter‐chromosomal rearrangement involving chromosomes 1 and 4 between *C. colocynthis* and the other three *Citrullus* species, *C. lanatus, C. amarus* and *C. mucosospermus* (Figure 1a; Figure S4). Good collinearity was found between the entire chromosome 4 of *C. colocynthis* and chromosome 8 of melon (Figure 1a), suggesting that *C. colocynthis* likely carried the ancestral karyotype and that the inferred chromosome fission and fusion events occurred approximately 4.54–2.41 Mya after the divergence of *C. colocynthis* from other watermelons and before the separation of *C. amarus* from *C. mucosospermus* and *C. lanatus* (Figure S5).

**Figure 1:**
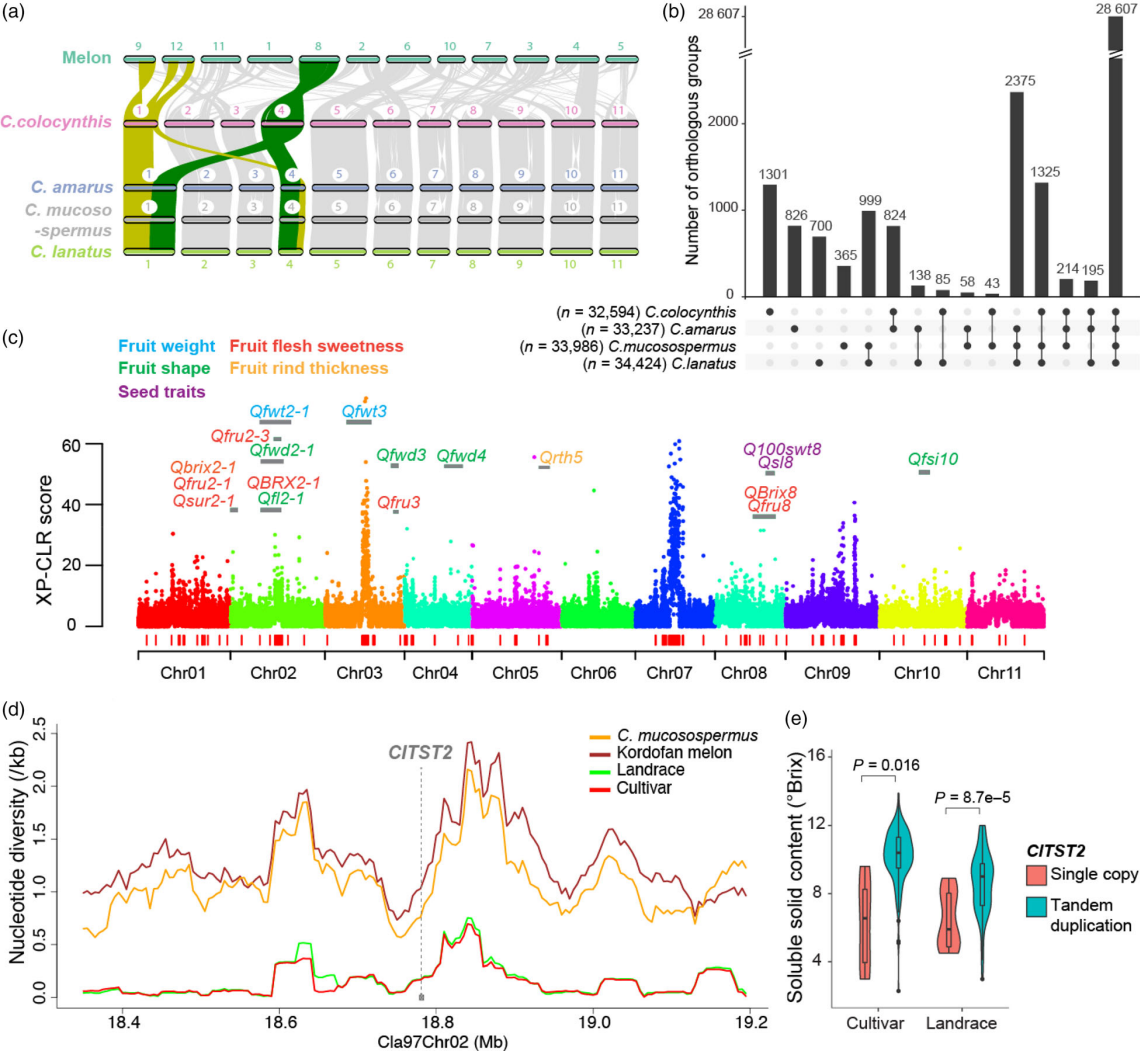
*Citrullus* genus super‐pangenome reveals variations in wild and cultivated watermelons. (a) Synteny among the genomes of melon and various watermelon species. Genomic regions syntenic to *C. colocynthis* chromosomes 1 and 4 are highlighted in yellow and green, respectively. (b) Upset diagram of orthologous groups among the four watermelon species. (c) Domestication sweeps detected through comparing landraces to Kordofan melons. Red bars at the bottom indicate genomic regions under selection. (d) Nucleotide diversities in genome regions surrounding the *ClTST2* gene in different watermelon populations. (e) Comparison of fruit flesh sweetness between accessions carrying the two different alleles of *ClTST2*, using *t*‐test with unequal variance.

We resequenced 201 wild watermelon accessions. Combined with the reference genomes and previously generated resequencing data (Guo *et al*., 2019), a total of 547 watermelon accessions were used in the watermelon super‐pangenome construction, including 349 *C. lanatus* (243 cultivars, 88 landraces and 18 *C. lanatus* subsp. *cordophanus*), 31 *C. mucosospermus*, 131 *C. amarus* and 36 *C. colocynthis* (Tables S6 and S7). Four species‐level pangenomes were first built (Figure S6a), each containing the species‐specific reference sequences, and a total of 24.5 Mb, 15.6 Mb, 18.3 Mb and 42.4 Mb non‐redundant novel sequences for *C. lanatus*, *C. mucosospermus*, *C. amarus* and *C. colocynthis*, respectively, harbouring 2288, 583, 1922 and 2521 novel genes that were absent in the species‐specific reference genomes (Table S8). The four species‐level pangenomes were combined into a *Citrullus* super‐pangenome based on orthologous relationships between genes from different species (gene to gene) and between aligned genes and genomic regions without predicted genes (gene to location) (Figure S6b; Appendix S1). As a result, 34 910 orthologous groups, including 33 697 syntenic orthologous groups, 1166 orthologous groups without syntenic information, and 47 species‐specific groups, as well as 3145 singletons, were obtained (Tables S9 and S10; Figure S7). A total of 28 607 (75.2%) orthologous groups contained genes or sequences from all four species (Figure 1b), among which 27 438 (95.9%) had one gene or one location in each species (1 : 1 : 1 : 1).

Resequencing reads of each watermelon accession were aligned to the pangenome of its own species for detecting gene presence/absence variations (PAVs). Accessions with insufficient read coverage were excluded to control false calls of gene PAVs. In the *Citrullus* super‐pangenome, the content of core genes (present in all accessions) was 63.7% (24 235 genes), much lower than that in the species‐level pangenomes (85.6%, 97.2%, 90.0% and 88.7% for *C. lanatus*, *C. mucosospermus*, *C. amarus* and *C. colocynthis*, respectively) (Figures S8–S10), indicating diverse genetic makeups among the four species. Genes with different occurrence frequencies between watermelon species or groups were identified (Tables S11–S14) and included those that were under selection during watermelon domestication and improvement (Table S15). 17 disease resistance‐related genes that were absent or present at low frequencies in the *C. lanatus* gene pool while present at high frequencies in at least one of the wild species gene pools were identified (Table S16).

We compared *C. lanatus* landrace to Kordofan melon (Figures S11 and S12), recently found to be the possible direct progenitor of cultivated watermelon (Renner *et al*., 2021), and identified 123 domestication sweeps with a cumulative length of 17.62 Mb (Table S17) and harbouring 399 annotated genes, among which 107 were in fruit quality QTLs (Figures 1c, S13 and S14; Table S18). The Kordofan melons were not sweet with flesh soluble solids content (SSC) ranging from 0.2 to 3.2 °Brix (Table S19). The sugar transporter ClTST2 regulates sugar accumulation in watermelon flesh (Ren *et al*., 2018). The Kordofan melon line having the highest SSC carried the *ClTST2* tandem duplication (Table S19; Figure S15; Appendix S1). A genetic diversity reduction was observed in *ClTST2* genomic region in landraces compared to Kordofan melon (Figure 1d). This *ClTST2* tandem duplication became a predominant allele in landraces (70 out of 86 accessions; 81.4%) and was almost fixed in cultivars (238 out of 245 accessions; 97.1%) (Table S20). Fruit flesh SSC levels were significantly higher in accessions carrying the *ClTST2* tandem duplication compared to the ones with only one copy (Figure 1e). These results together suggested that the *ClTST2* tandem duplication was present in wild watermelon populations and was selected during domestication likely due to its important role in promoting sugar accumulation in fruits.

Collectively, our *Citrullus* super‐pangenome provides insights into watermelon evolution and domestication and serves as a comprehensive resource for researchers and breeders to mine and utilize genes in cultivated and wild watermelon species.

## Conflict of interest

The authors declare no competing interest.

## Author contributions

Z.F. and S.W. designed and managed the project. S.B., W.P.W., C.K., A.L., C.M., S.S.R., X.Y. and Z.F. provided plant materials and/or contributed to DNA and RNA sequencing. S.H, S.W. and L.G. performed data analyses. S.W. and H.S. wrote the manuscript. Z.F. revised the manuscript.

## Supporting information


**Appendix S1** Supploementary notes and methods.

**Figure S1** K‐mer distribution of Illumina reads.
**Figure S2** Collinearity between the USVL246‐FR2 pseudomolecules and genetic maps.
**Figure S3** Hi‐C chromatin interaction heatmap of *C. colocynthis* PI 537277.
**Figure S4** Collinearity between watermelon genomes.
**Figure S5** Phylogeny and estimated times of divergence events.
**Figure S6** Workflow for *Citrullus* super‐pangenome construction.
**Figure S7** Genes in the *Citrullus* super‐pangenome.
**Figure S8** Compositions of the four species‐specific pan‐genomes.
**Figure S9** Compositions of the *Citrullus* super‐pangenomes.
**Figure S10** Numbers genes detected in individuals of different watermelon populations.
**Figure S11** Phylogenetic tree of wild and cultivated accessions.
**Figure S12** Mature fruits of 15 Kordofan melon accessions.
**Figure S13** Expression levels of *Cla97C05G101010*.
**Figure S14** Nucleotide diversities in *ClBt* (a) and *LCYB* (b) genomic regions.
**Figure S15** Read alignment at *ClTST2*.

**Table S1** Summary statistics of Illumina reads.
**Table S2** Summary statistics of de novo assemblies of three wild watermelon reference g.
**Table S3** Summary of pseudomolecule statistics.
**Table S4** Summary of repeat annotation.
**Table S5** BUSCO completeness scores of watermelon genome assemblies and annotations.
**Table S6** Watermelon accessions used in the pan‐genome study.
**Table S7** SNPs and small indels identified in watermelon.
**Table S8** Summary statistics of the species‐level pan‐genomes.
**Table S9** *Citrullus* super‐pangenome orthologous groups.
**Table S10** Orthologous groups specifically present or expanded in *C. lanatus*.
**Table S11** Number of genes with significantly different occurrence frequencies between populations.
**Table S12** Genes with significantly different occurrence frequencies between populations.
**Table S13** Enriched Biological Processes.
**Table S14** Genes related to meristem maintenance and development that had significantly higher frequencies in the *C. lanatus* and *C. mucosospermus*.
**Table S15** Genes with significantly changed occurrence frequencies during watermelon domestication and improvement.
**Table S16** Genes related to disease resistance that had significantly different frequencies in the cultivated watermelon compared to the wild species.
**Table S17** Putative domestication sweeps.
**Table S18** Genes in domestication sweeps.
**Table S19** Key fruit traits in Kordofan melons and genotypes at ClBt and LCYB genes.
**Table S20** Fruit flesh soluble solid content in watermelon accessions with different alleles for ClTST2.
