# Supplementary material for: A Citrullus genus super‐pangenome reveals extensive variations in wild and cultivated watermelons and sheds light on watermelon evolution and domestication
Source: Plant Biotechnol J. 2023 Jul 25;21(10):1926–8. doi: 10.1111/pbi.14120 (PMC10502741; doi:10.1111/pbi.14120)
Supplement: Supplementary file 1 — Appendix S1 Supploementary notes and methods. [file PBI-21-1926-s002.pdf]

Supplemental notes and methods for

A *Citrullus* genus super-pangenome reveals extensive variations in wild and cultivated watermelons and sheds light on watermelon evolution and domestication

De novo genome assembly and pseudochromosome construction

The PacBio reads of USVL531-MDR were assembled into contigs followed by polishing with PacBio and Illumina reads, which resulted in an assembly containing 77 contigs with a total size of 365.3 Mb and an N50 length of 27.6 Mb (**Table S2**). Based on the synteny to the cultivar 97103 reference genome, 99.36% of the USVL531-MDR assembly (363.0 Mb; 21 contigs) were anchored to 11 chromosomes (**Table S3**). Ten out of the 11 USVL531-MDR chromosomes were each composed of only one or two contigs.

The high-quality cleaned sequences produced from paired-end and mate-pair libraries covered approximately 284.2× and 370.1× of the USVL246-FR2 and PI 537277 genomes, respectively (**Table S1**). The genome assembly of USVL246-FR2 had a total size of 386.7 Mb, and consisted of 38,258 contigs and 1,422 scaffolds, with N50 sizes of 18.1 kb and 3.8 Mb, respectively (**Table S2**). Using genetic maps generated from the recombinant inbred line (RIL) and F_2 populations derived from a cross between USVL246-FR2 and USVL114 (Branham et al., 2019), 95.9% of the assembly was anchored to 11 linkage groups and 93.6% of the assembly was oriented (**Figure S2** and **Table S3**). The PI 537277 genome was assembled into 15,928 contigs and 1,536 scaffolds with N50 sizes of 44.9 kb and 1.6 Mb, respectively, and the total assembly size was 356.1 Mb (**Table S2**). About 99.7% of the PI 537277 assembled sequences were constructed into 11 pseudochromosomes using Hi-C data (**Figure S3** and **Table S3**). The GC contents of the USVL531-MDR, USVL246-FR2 and PI 537277 assemblies were 33.66%, 33.55% and 33.31%, respectively.

Quality evaluation of watermelon genome assemblies

Completeness of the three assemblies was evaluated by BUSCO (Simao et al., 2015), which revealed that 99.07%, 98.95% and 99.01% of the core conserved plant genes were detected complete in the USVL531-MDR, USVL246-FR2 and PI 537277 assemblies, respectively (**Table S5**). Aligning genomic reads back to the assembly using BWA-MEM (v0.7.17) (Li, 2013) revealed mapping rates of 98.58%, 98.72% and 99.67% for USVL531-MDR, USVL246-FR2 and PI

537277, respectively. The LAI values (Ou et al., 2018) were 10.66, 7.17 and 8.53 for USVL531-MDR, USVL246-FR2 and PI 537277, respectively, comparable to that of the high-quality 97103 reference genome (9.96; Guo et al., 2019). Collectively, these results indicated high quality of the three watermelon genome assemblies.

Divergence among watermelon species

Molecular dating using single-copy orthologous genes in genomes of the five watermelon species and subspecies, seven other cucurbit species, strawberry and walnut revealed that *Citrullus* species diverged from their sister clade 13.79 (10.30 to 17.39) million years ago (Mya) (**Figure S5**), consistent with the previous finding (Wu et al., 2017). The divergence between cultivated watermelon and wild Kordofan melon was dated to be 0.18 (0.12 to 0.24) Mya, and *C. lanatus* split from its West African sister, *C. mucosospermus*, 0.25 (0.17 to 0.32) Mya. The inferred divergence time between cultivated watermelon and its direct progenitor, Kordofan melon, was earlier than the anticipated time of watermelon domestication (Renner et al., 2021), and could be an overestimation caused by genes exhibiting incomplete lineage sorting due to the closely timed divergence events among *C. mucosospermus* and the two *C. lanatus* subspecies. Genome alignments between wild and cultivated watermelons revealed one-to-one chromosome-level syntenic relationships among *C. lanatus*, *C. mucosospermus* and *C. amarus*, with several large inversions (**Figure S4**).

Gene evolution in watermelon species

When keeping genomic regions without annotated genes but aligned by genes from other species, 28,607 (75.2%) orthologous groups contained genes or sequences from all four species (**Figure 1b**), among which 27,438 (95.9%) had one gene or one location in each species (1:1:1:1). However, when only considering gene-to-gene orthologous relationships, the proportion of species-specific genes increased from 8.4% (3,193 including species-specific groups and singletons) to 42.6% (16,217) (**Figure S7a**), with *C. colocynthis* having the most species-specific genes (**Figure S7b** and **S10b**), consistent with its most distant phylogenetic relationship to the other three watermelon species. These results together suggested that during watermelon evolution, mutations in genes such as those causing premature stops or frame shifts had occurred and possibly led to disruptions of gene functions as indicated by the absence of predicted gene models in those orthologous

genomic regions; however, the sequences derived from the ancestral genes were largely maintained in the genomes rather than being completely purged.

Genes that are unique to *C. lanatus* might include those underlying fruit quality traits that distinguish it from its wild relatives. There were 701 *C. lanatus*-specific orthologous groups and another 627 groups contained *C. lanatus*-specific tandem duplicated copies (at least two copies of *C. lanatus* genes in the syntenic region, while at most one was found in each of the other three species) (**Table S10**). These genes included the tonoplast sugar transporter gene, *ClTST2* (*Cla97C02G036390* and *Cla97C00G000440*), which functions in regulating sugar accumulation in watermelon flesh (Ren et al., 2018). This *ClTST2* tandem duplication has also been found in the elite watermelon line G42 (Deng et al., 2022).

The numbers of orthologous genes and genomic locations found in the four species ranged from 32,594 to 34,425. *C. colocynthis* and *C. amarus* individuals carried significantly fewer genes/loci on average than *C. mucosospermus* and *C. lanatus* individuals (**Figure 1b**). However, regarding the predicted genes, *C. colocynthis* had the most genes both at the species level and in individual genomes (**Figure S7b**). These results revealed differences in the loss of ancestral gene loci and the retention of functional genes in the genomes of various *Citrullus* species, likely due to lineage-specific evolutionary events. Fewer genes/loci were found in the genomes of cultivated individuals than in those of Kordofan melons (**Figure S10**), suggesting gene loss during watermelon domestication, a phenomenon that has also been reported in tomato (Gao et al., 2019).

Selection of genes during watermelon evolution and domestication

Genes with different occurrence frequencies between watermelon species or groups include those involved in adaptation and/or domestication. Pairwise comparisons among the four species were carried out and genes with significantly different presence frequencies (FDR < 0.001 and fold change > 2) between species were identified. About five to seven thousand such genes were found between any two of the four *Citrullus* species, except that only about one thousand genes with significantly altered frequencies were found between the closely related *C. lanatus* and *C. mucosospermus* (**Tables S11** and **S12**). For all species, when compared with another species, genes with significantly increased frequencies were enriched with biological processes related to "photosynthesis" and "nucleotide metabolic process" (**Table S13**). These results suggested that after speciation, different watermelons had kept different sets of genes participating in these most

basic biological processes in plants. This might be a result of random losses of functionally redundant genes or represent a fine-tuning of these pathways through actively retaining specific genes in response to the environmental conditions that different watermelon species were exposed to. Interestingly, in comparison to *C. colocynthis* and *C. amarus*, genes with increased frequencies in *C. mucosospermus* and *C. lanatus* were enriched with biological processes "meristem maintenance" and "meristem development". Of the 24 genes associated with these biological processes and with significantly higher frequencies in *C. mucosospermus* and *C. lanatus*, eighteen encoded serine/threonine-protein phosphatase 7 long form proteins (**Table S14**), orthologous to Arabidopsis *MAINTENANCE OF MERISTEMS LIKE 3* that is expressed in the vasculature and hydathodes of leaves (Ühlken et al., 2014). PAV of these genes may contribute to differences in leaf growth observed among watermelon species (Levi et al., 2017).

By comparing *C. lanatus* landrace to its progenitor, Kordofan melon, 145 genes were found to have significantly changed occurrence frequencies during domestication (130 and 15 with decreased and increased frequencies, respectively), while during improvement from landraces to cultivars, only 13 genes had significantly altered frequencies (11 decreased and 2 increased) (**Tables S11** and **S15**). Fruit quality traits including flesh sweetness and coloration changed dramatically during watermelon domestication and improvement. Our results suggested these changes might be achieved mainly through mutations modifying the function of existing genes or their expression patterns, rather than removal or introduction of genes. A gene encoding late embryogenesis abundant protein, *Cla97C06G117170*, had a decreased frequency in cultivated watermelon compared to Kordofan melon and co-located with QTLs controlling seed traits (**Table S15**). The potential function of *Cla97C06G117170* suggested its role in seed embryo development. This gene could be crucial for the survival of watermelons in the wild but might have become less important under cultivated conditions. There were other genes found in QTLs and/or abundantly expressed in developing fruits (**Tables S15**), yet their functions require further investigation.

Revisiting the domestication of watermelon

We previously used *C. mucosospermus* to represent the wild progenitor of cultivated watermelon for domestication sweep detection, due to its close phylogenetic relationship to *C. lanatus* and its primitive fruit flesh characteristics, such as the lack of both sweetness and coloration (Guo et al., 2019). In this study, we included 18 lines of Kordofan melon, a descendant of the possible direct

progenitor of cultivated watermelon (Renner et al., 2021). A total of 13,256,154 and 2,277,760 high-quality SNPs and small insertions/deletions (indels) were identified among the 547 watermelon accessions. Among the SNPs, 260,380 led to non-synonymous changes and 11,066 led to start/stop codon gain/loss, and 28,445 small indels caused coding sequence changes (**Table S7**). Phylogenetic analysis using SNPs at fourfold degenerate sites revealed that the 18 Kordofan melons formed a monophyletic clade that was more closely related to cultivated watermelons than any other wild species (**Figure S11**). The nucleotide diversity (π) in Kordofan melons (n = 18) was 0.84×10^{-3}, higher than that of landraces (n = 88; $\pi = 0.71\times10^{-3}$) and cultivars (n = 243; $\pi = 0.58\times10^{-3}$). These results together supported the current hypothesis that Kordofan melon in Northeast Africa is the progenitor of the cultivated watermelon rather than a feral form (Renner et al., 2021).

To study the watermelon genomic landscape altered by domestication, we compared *C. lanatus* landrace to the direct wild progenitor, Kordofan melon, and identified 123 domestication sweeps with a cumulative length of 17.62 Mb (**Table S17**) and harboring 399 annotated genes, among which 107 were in fruit quality QTLs, including eight controlling flesh sweetness on chromosomes 2, 3 and 8, two controlling fruit weight on chromosomes 2 and 3, and five controlling fruit shape on chromosomes 2, 3, 4 and 10 (**Figure 1c** and **Table S18**). One of these genes, *Cla97C05G101010*, encoding a cytochrome P450 CYP82D47-like protein, was not identified previously in domestication sweeps using *C. mucosospermus* as the progenitor (Guo et al., 2019), and was in a rind thickness QTL, *Qrth5* (Sandlin et al., 2012). Its high expression levels in the fruit rind of watermelon 97103 (**Figure S13**; Guo et al., 2013) made it a strong candidate gene for this trait. Bitterness of fruit flesh is variable in *C. mucosospermus*, and the non-bitter trait has been fixed in cultivated watermelon (Guo et al., 2019). None of the Kordofan melons were bitter and all of them carried the homozygous non-bitterness allele at the *ClBt* gene (**Table S19**), which contains a mutation leading to a premature stop codon and loss-of-function of the gene (Zhou et al., 2016). In contrast to *C. mucosospermus*, the genomic region surrounding *ClBt* had a low genetic diversity in Kordofan melon comparable to that in cultivated watermelon (**Figure S14a**), suggesting that loss of bitterness in fruit flesh had already happened in the progenitor of cultivated watermelon prior to domestication. Kordofan melons displayed white or pinkish fruit flesh colors (**Figure S12**) and carried different alleles at the SNP site in the *lycopene β-cyclase* (*LCYB*) gene, controlling red coloration of fruit flesh (**Table S19**), which leads to an amino acid change from a conserved phenylalanine to valine (Bang et al., 2007). Kordofan melons had kept higher genetic

diversity in the *LCYB* genomic regions in comparison to landraces and cultivars (**Figure S14b**), suggesting that *LCYB* has been selected during watermelon domestication and improvement.

Methods

Plant materials and sequencing

Three wild watermelon accessions were selected for reference genome sequencing: *C. mucosospermus* USVL531-MDR, resistant to powdery mildew and Phytophthora fruit rot (Mandal et al., 2020); *C. amarus* USVL246-FR2, resistant to Fusarium wilt (Wechter et al., 2016) and bacterial fruit blotch (Branham et al., 2019); and *C. colocynthis* PI 537277, resistant to whiteflies (Coffey et al., 2015) and Papaya ringspot virus-watermelon strain (Levi et al. 2016). Plants of *C. mucosospermus* USVL531-MDR were grown in the greenhouse at Boyce Thompson Institute in Ithaca, New York, with a 16/8 h light/dark cycle at 20°C (night) to 25°C (day). Young leaves from a single 3-week-old plant were collected for high-molecular-weight DNA extraction followed by shearing to fragments with an average size of 20 kb using g-TUBE (Covaris). The sheared DNA was then used to construct a PacBio SMRT library following the standard SMRT bell construction protocol. The library was sequenced on a PacBio Sequel platform using the 2.0 chemistry (PacBio). A paired-end genomic libraries with an insert size of 470 bp was prepared using the Genomic DNA Sample Prep kit (Illumina, San Diego, CA) and sequenced on an Illumina NextSeq 1000 platform. Plants of *C. colocynthis* PI 537277 and *C. amarus* USVL246-FR2 were grown in the greenhouse at the U.S. Vegetable Laboratory, Charleston, South Carolina, with 14-16 h of natural sun light and temperature at 25-30°C. Genomic DNA was extracted from young fresh leaves using the QIAGEN DNeasy Plant Mini Kit (QIAGEN, Valencia, CA) following the manufacturer's instructions. Paired-end genomic libraries with insert sizes of 200 bp and 500 bp for USVL246-FR2, and 470 bp and 800 bp for PI 537277 were prepared using the Genomic DNA Sample Prep kit (Illumina, San Diego, CA). Four mate-pair libraries with insert sizes of 5, 10 and 15 and 20 kb for USVL246-FR2 and three mate-pair libraries with 2, 5 and 10 kb insert sizes for PI 537277 were prepared. All these paired-end and mate libraries were sequenced on an Illumina HiSeq 1500 system with the paired-end mode. For PI 537277, Hi-C and Chicago libraries were prepared following the protocols implemented by Dovetail Genomics (Scotts Valley, CA, USA) and sequenced on an Illumina HiSeq X platform.

Transcriptome sequencing was performed for samples collected from *C. amarus*

USVL246-FR2 leaves and *C. colocynthis* PI 537277 fruit tissues. Total RNA was extracted using QIANGEN RNeasy Plant Mini Kit (QIANGEN). RNA-Seq libraries were constructed using the NEB Next UltraTM RNA Library Prep Kit (NEB, Beverly, MA) and sequenced on a HiSeq 1500 platform.

Fifteen Kordofan melon lines were grown in the greenhouse at Boyce Thompson Institute in Ithaca, New York, with a 16/8 h light/dark cycle and night and day temperature of 20°C and 25°C, respectively. Flowers were hand pollinated at anthesis and fruits were collected at 54 to 74 days after pollination for the measurement of soluble sugar content on a digital refractometer (HR200; APT Co.).

For genome resequencing, young fresh leaf tissue from a single seedling of a total of 202 watermelon accessions including 201 wild accessions and one landrace were collected. Genomic DNA was extracted from the leaf tissue using the Qiagen DNeasy Plant Kit, followed by paired-end library construction using the NEBNext Ultra DNA Library Prep kit according to the manufacturer's instructions. The libraries were sequenced on an Illumina NextSeq 1000 platform using the paired-end 2 × 150 bp mode.

***De novo* reference genome assembly**

PacBio reads of *C. mucosospermus* USVL531-MDR were error corrected and assembled into contigs using CANU (v1.7.1) (Koren et al. 2017) with default parameters except that both 'OvlMerThreshold' and 'corOutCoverage' were set to 500. The resulting contigs were then corrected with PacBio long reads using the Arrow program in the SMRT-link-5.1 package (PacBio). Illumina paired-end genome sequencing reads were processed with Trimmomatic (v0.36) (Bolger et al., 2014) to remove adaptors and low-quality sequences. The corrected assembly was subjected to a second-round error correction with the cleaned Illumina reads using Pilon (v1.22) (Walker et al., 2014) with parameters '-fix bases -diploid'. Putative contaminations were identified by aligning the assemblies against the NCBI GenBank nucleotide database. Sequences with > 90% of their length aligned to the microbial or organelle genomes were discarded. Redundant contigs that were covered by longer contigs with sequence identity > 99% and coverage > 99% were removed. To construct pseudochromosomes, the contigs were aligned to the 97103 (v2) reference genome using LAST (Kielbasa et al., 2011) to find unique best alignments between USVL531-MDR and 97103. The genetic map constructed using a population derived from a cross between *C. lanatus*

cultivar Strain II and *C. mucosospermus* PI 560023 (Sandlin et al., 2012) was used to validate the chromosome-level assembly.

For *C. colocynthis* PI 537277 and *C. amarus* USVL246-FR2, Illumina paired-end and mate-pair reads were used for *de novo* genome assemblies. Trimmomatic (v0.36) (Bolger et al., 2014) and ShortRead (Morgan et al., 2009) were used to remove low-quality and adaptor sequences in reads from the paired-end and mate-pair libraries, respectively. The cleaned reads were assembled into scaffolds with SOAPdenovo2 (Luo et al., 2012), followed by gap filling with the gapcloser program in the SOAPdenovo2 package. Pilon (v1.22) (Walker et al., 2014) was used to correct base errors, fix mis-assemblies and further fill gaps. Contamination and redundant sequences were removed as describe above for USVL531-MDR. For pseudochromosome construction of PI 537277, the scaffolds, cleaned Illumina paired-end reads, Chicago library reads, and Dovetail Hi-C library reads were used as input data for HiRise (Dovetail Genomics), a software pipeline designed for using proximity ligation data to scaffold genome assemblies. The assembly was then manually inspected and curated based on paired-end and mate-pair read alignments. USVL246-FR2 scaffolds were anchored into 11 chromosomes using genetic maps generated from the F2 and RIL populations derived from USVL246-FR2 × USVL114 (Branham et al., 2019).

Repeat annotation and gene prediction and annotation

Repeat annotation was performed using the EDTA pipeline (Ou et al., 2019). The resulting repeat libraries were used to identify TEs in the genome assemblies with repeatmasker (http://www.repeatmasker.org/). Gene prediction was performed on the repeat-masked genome assemblies with MAKER (Cantarel et al., 2008), which combines evidence from *ab initio* gene prediction, transcript mapping and protein homology to define confident gene models. SNAP (Korf, 2004) and AUGUSTUS (Stanke et al., 2006) were used for *ab initio* gene prediction. *De novo* and genome-guided transcript assemblies were generated with Trinity (Grabherr et al., 2011) using RNA-Seq data generated in this study for *C. amarus* USVL246-FR2 and *C. colocynthis* PI 537277, and previously published 97103 RNA-Seq data (NCBI SRA accession SRP012849) for *C. mucosospermus* USVL531-MDR. The two types of transcript assemblies were then combined and aligned to the genomes using the PASA2 pipeline (Haas et al., 2003). The resulting alignments were used as the transcript evidence. To provide protein homology evidence, protein sequences

from Arabidopsis (TAIR 10), watermelon (97103 v2), cucumber (Gy14 v2), and melon (DHL92 v4.0), as well as the UniProt (Swiss-Prot plant division) database were aligned to the genomes using Spaln (Iwata and Gotoh, 2012). Furthermore, gene predictions of the six watermelon reference genomes, including three reported in this study and three published ones, *C. lanatus* 97103 (Guo et al., 2019), *C. lanatus* Charleston Gray (Wu et al., 2019) and Kordofan melon (*C. lanatus* subsp. *cordophanus*) (Renner et al., 2021), were improved through mapping genes between assemblies with Liftoff (Shumate and Salzberg, 2021). Briefly, coding sequences (CDS) of genes predicted in the other five watermelon reference genomes were aligned to one target genome, requiring one-to-one mapping with at least 90% identity and 90% coverage. Gene models originally predicted in the target genome and those 'lifted over' from the other five genomes were integrated, with the longest gene model kept for each gene. When adding a new gene model to a genomic region based on the Liftoff results, only non-TE genes containing InterPro domains were considered.

For gene annotation, protein sequences of the predicted genes were compared against the GenBank nr, the UniProt (Swiss-Prot and TrEMBL; http://www.uniprot.org/) and Arabidopsis (TAIR 10) databases using BLAST (Camacho et al., 2009) with an E-value cut-off of 1e-4, as well as the InterPro database using InterProScan (Jones et al., 2014). Gene ontology (GO) annotations were obtained using Blast2GO (Conesa et al., 2005) based on the BLAST results against nr and the InterProScan analysis. BLAST results against UniProt and TAIR were processed using AHRD (https://github.com/groupschoof/AHRD) for assigning functional descriptions to the predicted genes.

Divergence time and genome evolution

To estimate the divergence times among *Citrullus* species, genes in the genomes of five watermelons (*Citrullus lanatus* subsp. *vulgaris*, *C. lanatus* subsp. *cordophanus*, *C. mucosospermus*, *C. amarus*, and *C. colocynthis*), seven other cucurbit species (bottle gourd, bitter gourd, cucumber, melon, monk fruit, snake gourd, and sponge gourd), walnut (Zhang et al., 2020) and woodland strawberry (Edger et al., 2018) were used to perform molecular dating. Gene sequences of the cucurbit species were downloaded from CuGenDBv2 (Yu et al., 2023). CDS sequences of single-copy orthologous genes identified by OrthoFinder (v 2.5.4) (Emms and Kelly, 2019) were concatenated and aligned using MUSCLE (v5) (Edgar, 2004). Bayesian divergence

time estimation was performed using the MCMCtree program in PAML (v 4.10.5) (Yang, 2007) with the known divergence time between walnut and Cucurbitaceae (84-105 Mya) as the calibration node, 20,000 burn-in iterations, 500 sampling frequency, and 20,000 accepted samples. Chromosome synteny comparison was performed with MCScan (https://github.com/tanghaibao/jcvi/wiki/MCscan-(Python-version)) using gene sequences of melon (DHL92 v4.0), *C. colocynthis* PI 537377, *C. amarus* USVL246-FR2, *C. mucosospermus* USVL531-MDR and *C. lanatus* 97103.

Construction and annotation of watermelon species-level pan-genomes

Raw Illumina reads were processed to trim adapters and low-quality sequences using Trimmomatic (v0.36) (Bolger et al., 2014). The high-quality cleaned Illumina reads from each accession were *de novo* assembled using SPAdes (v3.12.0) (Bankevich et al., 2012) with parameters '--careful -k 55,77,99,127'. Assembled contigs with length ≥500 bp were kept. The assembled contigs were aligned to the reference genome of the same species using QUAST (v5.0.2) (Gurevich et al., 2013) with parameters '--min-identity 90.0 --min-alignment 300'. Genomes of 97103, USVL531-MDR, USVL246-FR2, and PI 537277 were used as the references for species *C. lanatus*, *C. mucosospermus*, *C. amarus*, and *C. colocynthis*, respectively. Unaligned sequences with length ≥500 bp were extracted and searched against the NCBI GenBank nucleotide database using BLAST (Camacho et al., 2009) to identify possible contaminations. Sequences with best hits from outside the green plants, or covered by known plant chloroplast or mitochondrial genomes, were removed. The cleaned non-reference sequences from all accessions of the species were combined and consolidated into unique sequences using CD-HIT (Li and Godzik, 2006) with an identity threshold of 0.9. Redundancy was further removed by performing all-versus-all alignments using BLAST with an identity threshold of 90%. The resulting cleaned non-redundant non-reference sequences and the reference sequences were merged as the species-level pan-genome. Protein-coding genes were predicted from non-reference sequences as described above for the reference genome assemblies.

Construction of the *Citrullus* super-pangenome

To build the *Citrullus* super pan-genome, orthologous relationships were established among the species-level pan-genome genes. CDS of genes in one species-level pan-genome were mapped to

the other three pan-genomes to determine the orthologous genes or genomic regions using Liftoff (Shumate and Salzberg, 2021), requiring one-to-one alignment with both identity and coverage ≥90%. Gene pairs with at least 50% overlapped CDS regions were considered as orthologous genes. Otherwise, the mapped genomic locations were recorded as the orthologous regions. The gene-to-gene and gene-to-location orthologous relationships identified from the pairwise bidirectional gene 'lifting over' were consolidated into orthologous clusters containing the four species. Singleton genes that could be aligned to the sequences in the orthologous clusters using Blat (Kent, 2002) with at least 95% of identity and 50% of coverage were further added to the existing orthologous groups. Remaining singletons were aligned to the pan-genomes using Blat (Kent, 2002), requiring a minimal identity of 95% and a minimal coverage of 90% to identify additional gene-to-location relationships. Syntenic genomic regions across species were determined based on the syntenic gene blocks identified using MCScanX (Wang et al., 2012) with gene anchors whose CDS sequences were reciprocal best hits between species. Based on the syntenic genomic regions, the original orthologous groups were then categorized into syntenic orthologous groups and orthologous groups without syntenic information.

Gene PAV analysis

Genomic reads from each accession were aligned to the pan-genome of its own species using BWA-MEM (v0.7.17) (Li, 2013) with default parameters. The read coverage of the pan-genome was then computed from the alignments using the 'genomecov' utility in the BEDTools suite (Quinlan and Hall, 2010) with parameters '-bg -split'. To control false negative calling of gene PAVs, accessions with sequences covering less than 320 Mb of the pan-genome with at least one read were excluded from the gene PAV analysis. Read coverages for genes were calculated using BEDTools 'intersect' with the parameter '-wao' based on gene positions. A gene with 50% of its CDS length covered by one read was considered as present in the accession. Genes with significantly changed occurrence frequencies between two groups were determined using the Fisher's exact test with FDR corrected for multiple comparisons.

For genotyping at *ClTST2* in watermelon accessions, a junction site uniquely present in the tandem duplication allele (in the 97103 reference) was identified. Illumina paired-end reads were aligned to the 97103 reference using BWA-ALN (v0.7.17) (Li, 2013) with parameters '-n 0.01 -o 1 -e 2' and 'bwa sampe -s'. Read alignment supporting the presence of the junction site were used

to indicate the presence of the *ClTST2* tandem duplication (**Figure S15**). Read counts were also calculated for a nearby genomic site (500 bp upstream of the junction site) as a control. Read alignments were found at the control site for all accessions in this study, and the lack of read support at the junction site was considered absence of tandem duplication or single-copy *ClTST2*.

Variant calling, phylogenetic analysis and selective sweep identification

Raw Illumina DNA reads were processed using Trimmomatic (v0.36) (Bolger et al., 2014) to remove low quality and adaptor sequences. The cleaned reads were aligned to the 97103 genome using BWA-MEM (v0.7.17) (Li, 2013) with default parameters. SNP and small indel calling was performed using the Sentieon software package (https://www.sentieon.com/). Briefly, duplicated read pairs in each alignment file were marked using the Sentieon Dedup function, and variants from each sample were then called with the Sentieon Haplotyper function, followed by joint variant calling using Sentieon GVCFtyper. Hard filtering was applied to the raw variant set using GATK (v4.1) (McKenna et al., 2010), with parameters 'QD < 2.0 || FS > 60.0 || MQ < 40.0 || MQRankSum < -12.5 || ReadPosRankSum < -8.0' for SNPs and ‘QD<2.0 || FS>200.0 || ReadPosRankSum <−20.0’ for small indels. Only bi-allelic variants with minor allele frequency (MAF) > 0.01 were used in the downstream analyses. Variants were annotated using SnpEff (v5.0e) (Cingolani et al., 2012).

Phylogenetic relationships among the 547 watermelon accessions were inferred using 106,430 SNPs at the fourfold degenerate sites. A maximum likelihood tree was constructed using IQ-TREE (v1.6.12) (Nguyen et al., 2015) with 1,000 bootstrap replicates and a *C. naudinianus* accession, PI 596694, as the outgroup. Cross-population composite likelihood ratio test was performed using XP-CLR (v1.0) (Chen et al., 2010) to scan the 97103 genome for domestication sweeps by comparing landraces to Kordofan melons with parameters ‘-w1 0.0005 100 100 1 -p0 0.7’. Genetic distances between adjacent SNPs were calculated based on physical distance of adjacent markers in an integrated genetic map (Ren et al., 2014). Genomic regions with XP-CLR scores in the top 10% overlapping with top 50% of π ratios of Kordofan melons to landraces were identified as potential selective sweeps.

Data availability

This Whole Genome assemblies been deposited at DDBJ/ENA/GenBank under the accessions

JAKCFO000000000, JALMEV000000000 and QLSR00000000 for *C. mucosospermus* USVL531-MDR, *C. amarus* USVL246-FR2, and *C. colocynthis* PI 537277, respectively. Raw genome and transcriptome sequencing reads have been deposited in the NCBI BioProject database under the accession numbers PRJNA476359, PRJNA808410 and PRJNA794184. Genome (http://cucurbitgenomics.org/v2/genome_summary) and pan-genome (http://cucurbitgenomics.org/v2/ftp/pan-genome/watermelon/super-pangenome/) assemblies and annotation, and SNPs and small indels in VCF file format (http://cucurbitgenomics.org/v2/ftp/reseq/watermelon/v3/) are also available at CuGenDBv2 (Yu et al., 2023).

References

Bang, H., Kim, S., Leskovar, D. & King, S. Development of a codominant CAPS marker for allelic selection between canary yellow and red watermelon based on SNP in lycopene β-cyclase (LCYB) gene. *Mol. Breed.* **20**, 63–72 (2007).

Bankevich, A. *et al.* SPAdes: a new genome assembly algorithm and its applications to single-cell sequencing. *J. Comput. Biol.* **19**, 455–477 (2012).

Bayer, P. E., Golicz, A. A., Scheben, A., Batley, J. & Edwards, D. Plant pan-genomes are the new reference. *Nat. Plants* **6**, 914–920 (2020).

Bolger, A. M., Lohse, M. & Usadel, B. Trimmomatic: a flexible trimmer for Illumina sequence data. *Bioinformatics* **30**, 2114–2120 (2014).

Branham, S. E., Levi, A. & Wechter, W. P. QTL mapping identifies novel source of resistance to fusarium wilt race 1 in *Citrullus amarus*. *Plant Dis.* **103**, 984–989 (2019).

Branham, S. E., Levi, A., Katawczik, M. L., & Wechter, W. P. QTL mapping of resistance to bacterial fruit blotch in *Citrullus amarus*. *Theor. and Appl. Genet.*, **132**, 1463-1471 (2019).

Camacho, C. *et al.* BLAST+: architecture and applications. *BMC Bioinformatics* **10**, (2009).

Cantarel, B. L. *et al.* MAKER: An easy-to-use annotation pipeline designed for emerging model organism genomes. *Genome Res.* **18**, 188–196 (2008).

Castanera, R., Ruggieri, V., Pujol, M., Garcia-Mas, J. & Casacuberta, J. M. An improved melon reference genome with single-molecule sequencing uncovers a recent burst of transposable elements with potential impact on genes. *Front. Plant Sci.* **10**, 1815 (2020).

Chen, H., Patterson, N. & Reich, D. Population differentiation as a test for selective sweeps. *Genome Res.* **20**, 393–402 (2010).

Cingolani, P. *et al.* A program for annotating and predicting the effects of single nucleotide polymorphisms, SnpEff: SNPs in the genome of *Drosophila melanogaster* strain w1118; iso-2; iso-3. *Fly* **6**, 80–92 (2012).

Coffey, J. L., Simmons, A. M., Merle Shepard, B., Tadmor, Y. & Levi, A. Potential sources of whitefly (Hemiptera: Aleyrodidae) resistance in desert watermelon (*Citrullus colocynthis*) germplasm. *HortScience* **50**, 13–17 (2015).

Conesa, A. *et al.* Blast2GO: a universal tool for annotation, visualization and analysis in functional genomics research. *Bioinformatics* **21**, 3674–3676 (2005).

Della Coletta, R., Qiu, Y., Ou, S., Hufford, M.B. & Hirsch, C.N. How the pan-genome is changing crop genomics and improvement. *Genome Biol.* **22,** 3 (2021).

Deng, Y. *et al.* A telomere-to-telomere gap-free reference genome of watermelon and its mutation library provide important resources for gene discovery and breeding. *Mol. Plant* **15**, 1268–1284 (2022).

Edgar, R. C. MUSCLE: multiple sequence alignment with high accuracy and high throughput. *Nucleic Acids Res.* **32**, 1792–1797 (2004).

Edger, P. P. *et al.* Single-molecule sequencing and optical mapping yields an improved genome of woodland strawberry (*Fragaria vesca*) with chromosome-scale contiguity. *Gigascience* **7**, 1–7 (2018).

Emms, D. M. & Kelly, S. OrthoFinder: Phylogenetic orthology inference for comparative genomics. *Genome Biol.* **20**, 238 (2019).

Grabherr, M. G. *et al.* Full-length transcriptome assembly from RNA-Seq data without a reference genome. *Nat. Biotechnol.* **29**, 644–652 (2011).

Guo, S. *et al.* The draft genome of watermelon (*Citrullus lanatus*) and resequencing of 20 diverse accessions. *Nat. Genet.* **45**, 51–58 (2013).

Guo, S. *et al.* Resequencing of 414 cultivated and wild watermelon accessions identifies selection for fruit quality traits. *Nat. Genet.* **51**, 1616–1623 (2019).

Gurevich, A., Saveliev, V., Vyahhi, N. & Tesler, G. QUAST: quality assessment tool for genome assemblies. *Bioinformatics* **29**, 1072–1075 (2013).

Haas, B. J. *et al.* Improving the Arabidopsis genome annotation using maximal transcript alignment assemblies. *Nucleic Acids Res.* **31**, 5654–5666 (2003).

Hufford, M. B. *et al.* De novo assembly, annotation, and comparative analysis of 26 diverse maize genomes.

Science **373**, 655–662 (2021).
Iwata, H. & Gotoh, O. Benchmarking spliced alignment programs including Spaln2, an extended version of Spaln that incorporates additional species-specific features. *Nucleic Acids Res.* **40**, e161 (2012).
Jones, P. *et al.* InterProScan 5: genome-scale protein function classification. *Bioinformatics* **30**, 1236–1240 (2014).
Kent, W. J. BLAT—The BLAST-like alignment tool. *Genome Res.* **12**, 656 (2002).
Khan, A. W. *et al.* Super-pangenome by integrating the wild side of a species for accelerated crop improvement. *Trends Plant Sci.* **25**, 148–158 (2020).
Kielbasa, S. M., Wan, R., Sato, K., Horton, P. & Frith, M. C. Adaptive seeds tame genomic sequence comparison. *Genome Res.* **21**, 487–493 (2011).
Koren, S. *et al.* Canu: scalable and accurate long-read assembly via adaptive k-mer weighting and repeat separation. *Genome Res.* **27**, 722–736 (2017).
Korf, I. Gene finding in novel genomes. *BMC Bioinformatics* **5**, 59 (2004).
Levi, A. *et al*. Resistance to Papaya ringspot virus-watermelon strain (PRSV-W) in the desert watermelon *Citrullus colocynthis*. HortScience 51:4–7 (2016).
Levi, A. *et al.* Genetic diversity in the desert watermelon *Citrullus colocynthis* and its relationship with *Citrullus* species as determined by high-frequency oligonucleotides-targeting active gene markers. *J. Am. Soc. Hortic. Sci.* **142**, 47–56 (2017).
Li, H. Aligning sequence reads, clone sequences and assembly contigs with BWA-MEM. Preprint at arXiv https://arxiv.org/abs/1303.3997 (2013).
Li, W. & Godzik, A. Cd-hit: a fast program for clustering and comparing large sets of protein or nucleotide sequences. *Bioinformatics* **22**, 1658–1659 (2006).
Luo, R. *et al.* SOAPdenovo2: an empirically improved memory-efficient short-read de novo assembler. *Gigascience* **1**, 18 (2012).
Mandal, M. K., Suren, H. & Kousik, C. Elucidation of resistance signaling and identification of powdery mildew resistant mapping loci (*ClaPMR2*) during watermelon-*Podosphaera xanthii* interaction using RNA-Seq and whole-genome resequencing approach. *Sci. Reports 2020 101* **10**, 14038 (2020).
McKenna, A. *et al.* The genome analysis toolkit: a MapReduce framework for analyzing next-generation DNA sequencing data. *Genome Res.* **20**, 1297–1303 (2010).
Morgan, M. *et al.* ShortRead: A bioconductor package for input, quality assessment and exploration of high-throughput sequence data. *Bioinformatics* **25**, 2607–2608 (2009).
Nguyen, L.-T., Schmidt, H. A., von Haeseler, A. & Minh, B. Q. IQ-TREE: A fast and effective stochastic algorithm for estimating maximum-likelihood phylogenies. *Mol. Biol. Evol.* **32**, 268–274 (2015).
Ou, S. J., Chen, J. F. & Jiang N. Assessing genome assembly quality using the LTR Assembly Index (LAI). *Nucleic Acids Res*. **46**, e126 (2018).
Ou, S. *et al.* Benchmarking transposable element annotation methods for creation of a streamlined, comprehensive pipeline. *Genome Biol.* **20**, 275 (2019).
Paris, H. S. Origin and emergence of the sweet dessert watermelon, *Citrullus lanatus*. *Ann. Bot.* **116**, 133–148 (2015).
Quinlan, A. R. & Hall, I. M. BEDTools: a flexible suite of utilities for comparing genomic features. *Bioinformatics* **26**, 841–842 (2010).
Ren, Y. *et al.* A tonoplast sugar transporter underlies a sugar accumulation QTL in watermelon. *Plant Physiol.* **176**, 836–850 (2018).
Ren, Y. *et al.* An integrated genetic map based on four mapping populations and quantitative trait loci associated with economically important traits in watermelon (*Citrullus lanatus*). *BMC Plant Biol.* **14**, 33 (2014).
Renner, S. S. *et al.* A chromosome-level genome of a Kordofan melon illuminates the origin of domesticated watermelons. *Proc. Natl. Acad. Sci. U. S. A.* **118**, e2101486118 (2021).
Sandlin, K. *et al.* Comparative mapping in watermelon [*Citrullus lanatus* (Thunb.) Matsum. et Nakai]. *Theor. Appl. Genet.* **125**, 1603–1618 (2012).

Shang, L. *et al.* A super pan-genomic landscape of rice. *Cell Res.* **32**, 878–896 (2022).
Shumate, A. & Salzberg, S. L. Liftoff: accurate mapping of gene annotations. *Bioinformatics* **37**, 1639–1643 (2021).
Simão, F. A., Waterhouse, R. M., Ioannidis, P., Kriventseva, E. V. & Zdobnov, E. M. BUSCO: assessing genome assembly and annotation completeness with single-copy orthologs. *Bioinformatics* **31**, 3210–3212 (2015).
Stanke, M., Tzvetkova, A. & Morgenstern, B. AUGUSTUS at EGASP: using EST, protein and genomic alignments for improved gene prediction in the human genome. *Genome Biol.* **7**, S11 (2006).
Ühlken, C., Horvath, B., Stadler, R., Sauer, N. & Weingartner, M. MAIN-LIKE1 is a crucial factor for correct cell division and differentiation in *Arabidopsis thaliana*. *Plant J.* **78**, 107–120 (2014).
Walker, B. J. *et al.* Pilon: An integrated tool for comprehensive microbial variant detection and genome assembly improvement. *PLoS One* **9**, e112963 (2014).
Wang, Y. *et al.* MCScanX: a toolkit for detection and evolutionary analysis of gene synteny and collinearity. *Nucleic Acids Res.* **40**, e49 (2012).
Wechter, W. P., McMillan, M. M., Farnham, M. W. & Levi, A. Watermelon germplasm lines USVL246-FR2 and USVL252-FR2 tolerant to *Fusarium oxysporum* f. sp. *niveum* race 2. *HortScience* **51**, 1065–1067 (2016).
Wu, S. *et al.* The bottle gourd genome provides insights into Cucurbitaceae evolution and facilitates mapping of a *Papaya ring-spot virus* resistance locus. *Plant J.* **92**, 963–975 (2017).
Wu, S. *et al.* Genome of 'Charleston Gray', the principal American watermelon cultivar, and genetic characterization of 1,365 accessions in the U.S. National Plant Germplasm System watermelon collection. *Plant Biotechnol.* **17**, 2246-2258 (2019).
Yang, Z. PAML 4: Phylogenetic analysis by maximum likelihood. *Mol. Biol. Evol.* **24**, 1586–1591 (2007).
Yu, J. *et al.* CuGenDBv2: an updated database for cucurbit genomics. *Nucleic Acids Res.* **1**, (2023).
Zhang, J. *et al.* A high-quality walnut genome assembly reveals extensive gene expression divergences after whole-genome duplication. *Plant Biotechnol. J.* **18**, 1848–1850 (2020).
Zhou, Y. *et al.* Convergence and divergence of bitterness biosynthesis and regulation in Cucurbitaceae. *Nat. plants* **2**, 16183 (2016).
