# Supplementary material for: A Citrullus genus super‐pangenome reveals extensive variations in wild and cultivated watermelons and sheds light on watermelon evolution and domestication
Source: Plant Biotechnol J. 2023 Jul 25;21(10):1926–8. doi: 10.1111/pbi.14120 (PMC10502741; doi:10.1111/pbi.14120)
Supplement: Supplementary file 2 — Figure S1 K‐mer distribution of Illumina reads. Figure S2 Collinearity between the USVL246‐FR2 pseudomolecules and genetic maps. Figure S3 Hi‐C chromatin interaction heatmap of C. colocynthis PI 537277. Figure S4 Collinearity between watermelon genomes. Figure S5 Phylogeny and estimated times of divergence events. Figure S6 Workflow for Citrullus super‐pangenome construction. Figure S7 Genes in the Citrullus super‐pangenome. Figure S8 Compositions of the four species‐specific pan‐genomes. Figure S9 Compositions of the Citrullus super‐pangenomes. Figure S10 Numbers genes detected in individuals of different watermelon populations. Figure S11 Phylogenetic tree of wild and cultivated accessions. Figure S12 Mature fruits of 15 Kordofan melon accessions. Figure S13 Expression levels of Cla97C05G101010. Figure S14 Nucleotide diversities in ClBt (a) and LCYB (b) genomic regions. Figure S15 Read alignment at ClTST2. [file PBI-21-1926-s001.pdf]

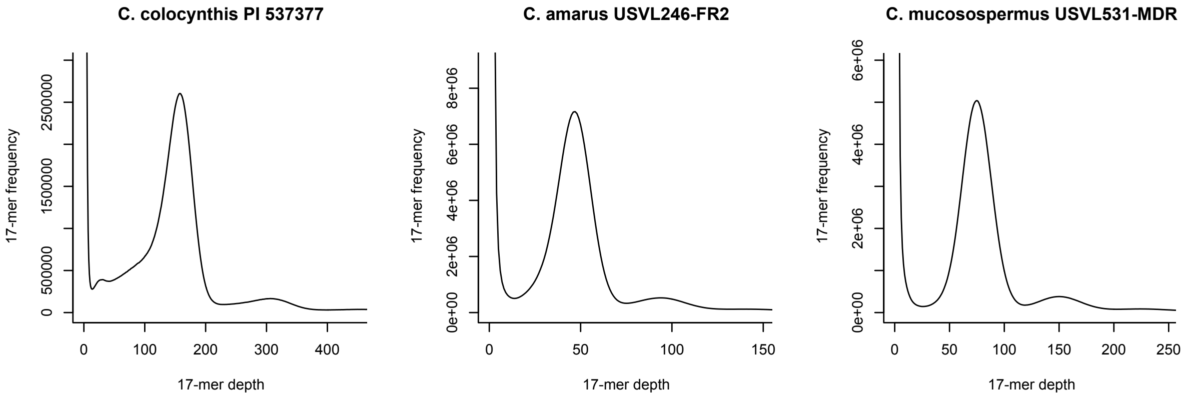

Figure S1 K-mer distribution of Illumina genomic sequencing reads of *C. colocynthis* PI 537277, *C. amarus* USVL246-FR2, and *C. mucosospermus* USVL531-MDR. Genome sizes of USVL531-MDR, USVL246-FR2 and PI 537277 were estimated to be 434.7 Mb, 423.2 Mb and 406.0 Mb, respectively. K-mer (k=17) counting was performed with Jellyfish (https://github.com/gmarcais/Jellyfish) and the genome size was estimated based on the formula: Genome size = total number of k-mers / peak depth.

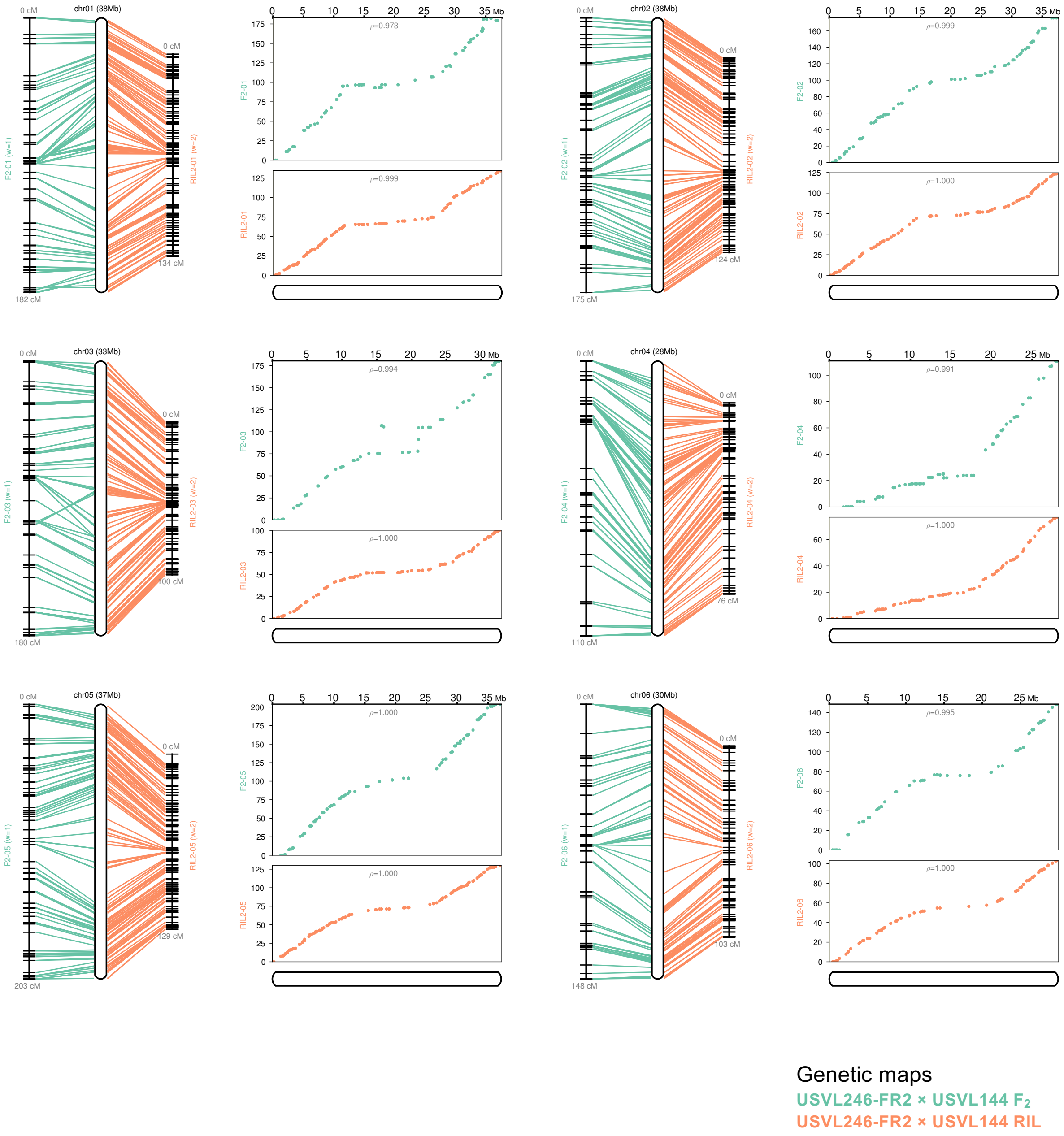

Figure S2 Collinearity between the USVL246-FR2 pseudomolecules and genetic maps.

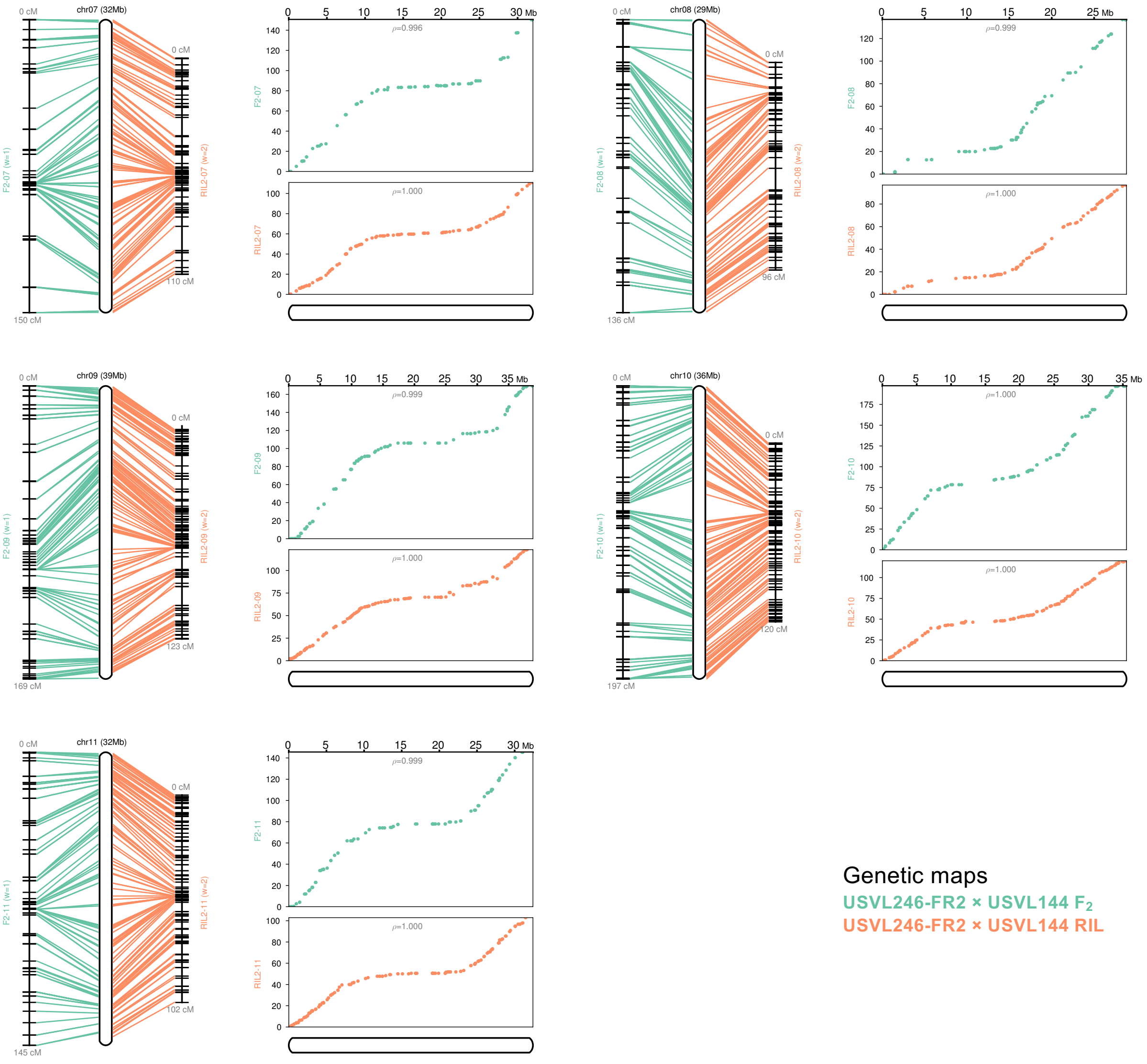

Figure S2 (Continued) Collinearity between the USVL246-FR2 pseudomolecules and genetic maps.

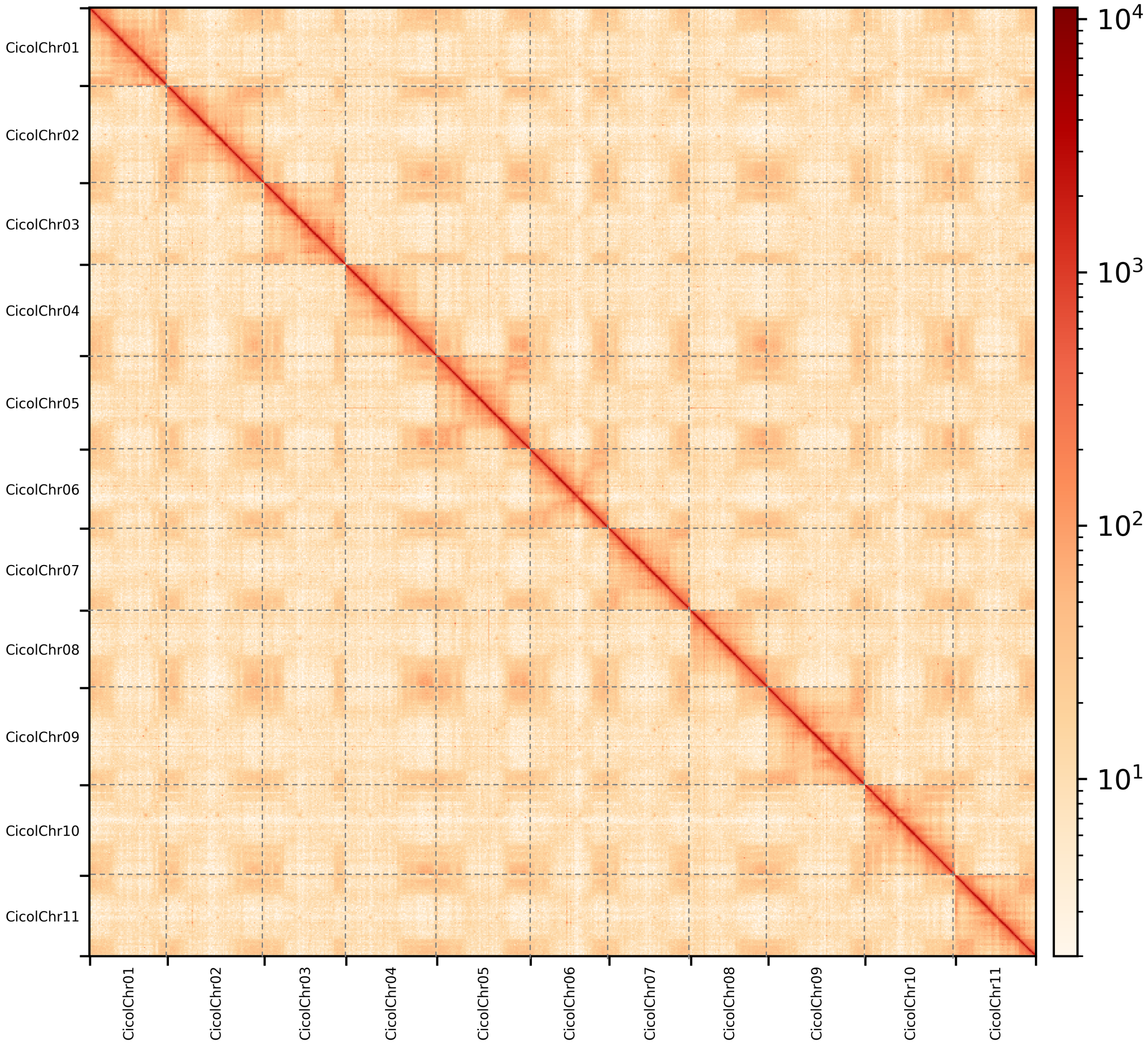

Figure S3 Hi-C chromatin interaction heatmap of *C. colocynthis* PI 537277.

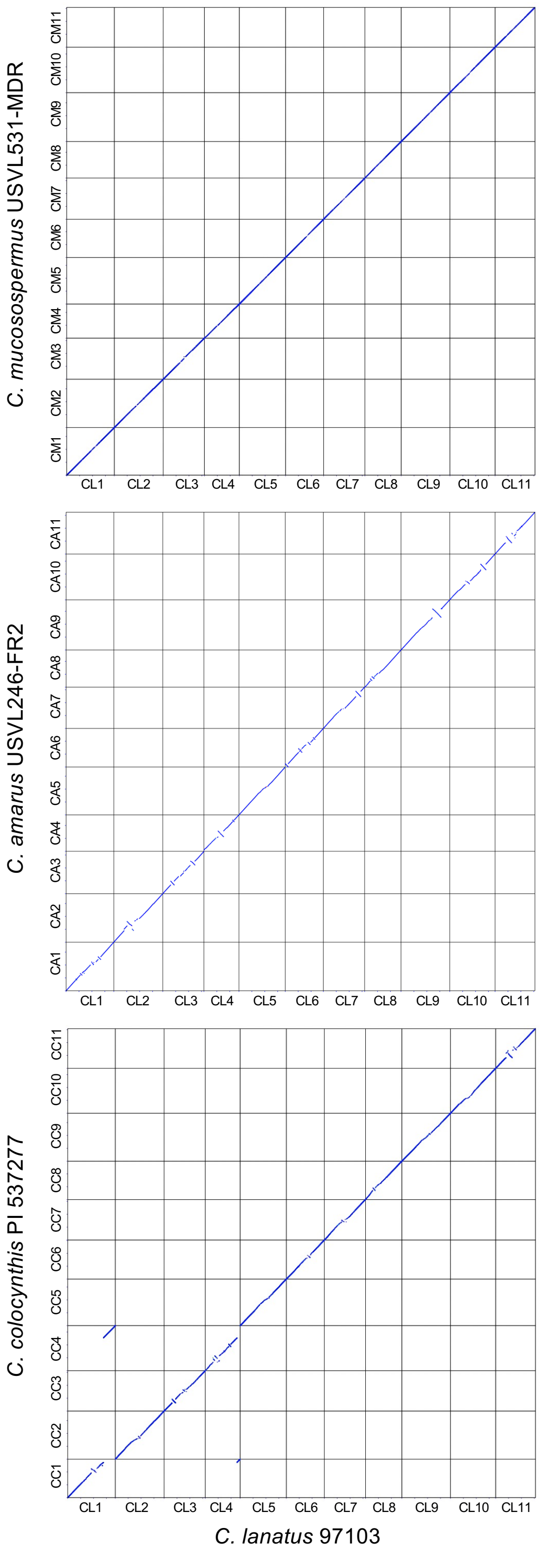

Figure S4 Collinearity between genomes of the cultivated watermelon 97103 and wild watermelons *C. mucosospermus* (CM) USVL531-MDR, *C. amarus* (CA) USVL246-FR2, and *C. colocynthis* (CC) PI 537277.

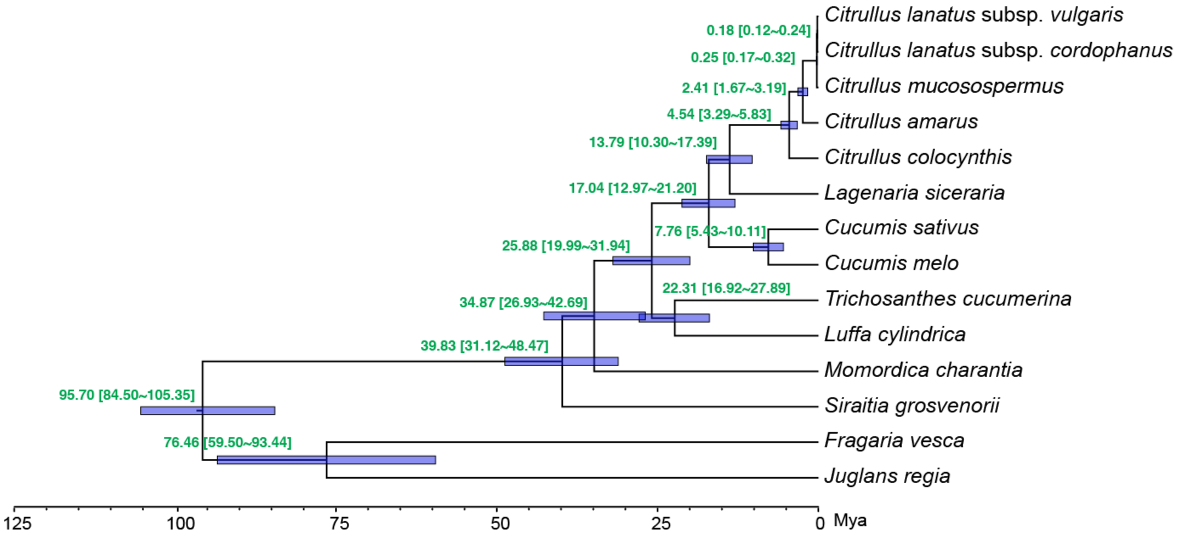

Figure S5 Phylogenetic tree and estimated times of divergence events. Green numbers near each node indicate the estimated divergence time in millions of years and the range of the 95% highest probability density intervals. Horizontal blue bar on each node indicates the range of the estimated divergence time. Genomes of five watermelons (*Citrullus lanatus* subsp. *vulgaris*, *C. lanatus* subsp. *cordophanus*, *C. mucosospermus*, *C. amarus* and *C. colocynthis*), bottle gourd (*Lagenaria siceraria*), cucumber (*Cucumis sativus*), melon (*Cucumis melo*), snake gourd (*Trichosanthes cucumerina*), sponge gourd (*Luffa cylindrica*), bitter gourd (*Momordica charantia*), monk fruit (*Siraitia grosvenorii*), woodland strawberry (*Fragaria vesca*) and walnut (*Juglans regia*) were used in the analysis.

(a)

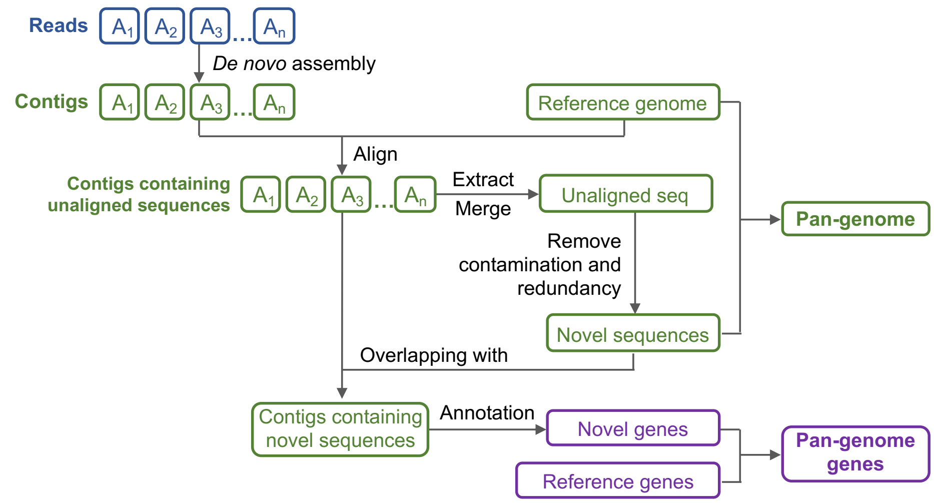

(b)

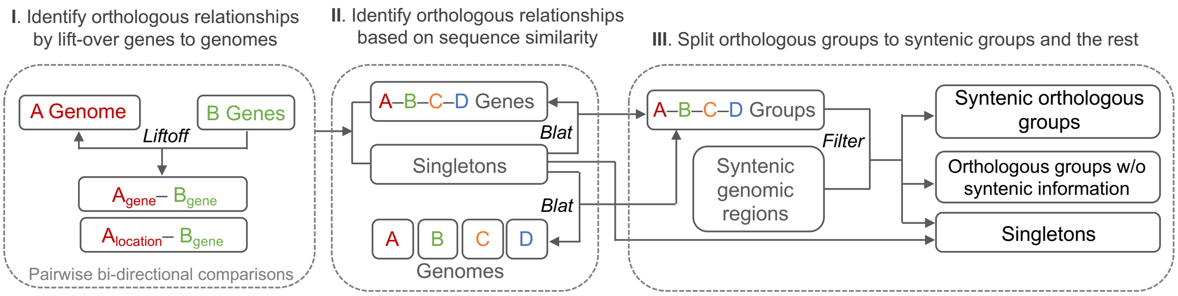

Figure S6 Workflow for the construction of the *Citrullus* super-pangenome. (a) Strategy for construction of species-level pan-genome by aligning *de novo* assembled sequences of individual accessions (A1, A2, A3, …, An) to the species-specific reference genome followed by identification of non-redundant novel sequences. (b) Strategy for construction of the *Citrullus* super-pangenome through establishing gene-to-gene and gene-to-location orthologous relationship among species-level pan-genomes. The colored A, B, C and D letters represent four *Citrullus* species.

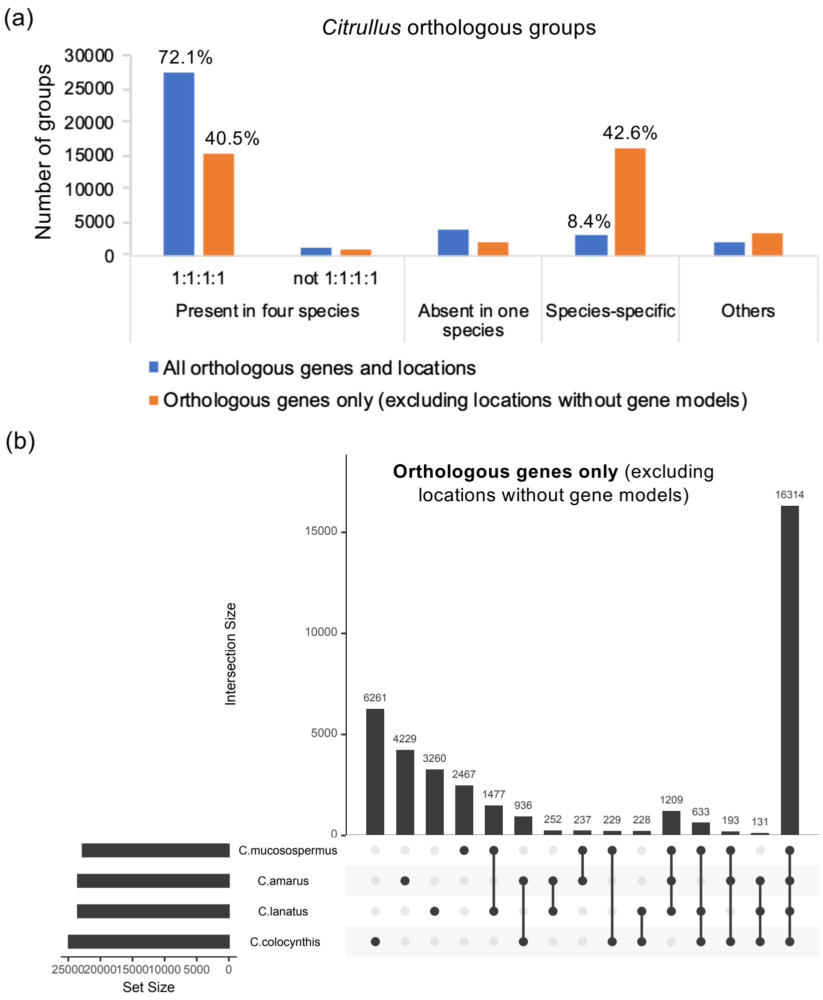

Figure S7 Genes in the *Citrullus* super-pangenome. (a) Proportions of shared and species-specific orthologous groups. (b) Upset diagram of orthologous gene groups among the four watermelon species, with orthologous locations without predicted genes excluded.

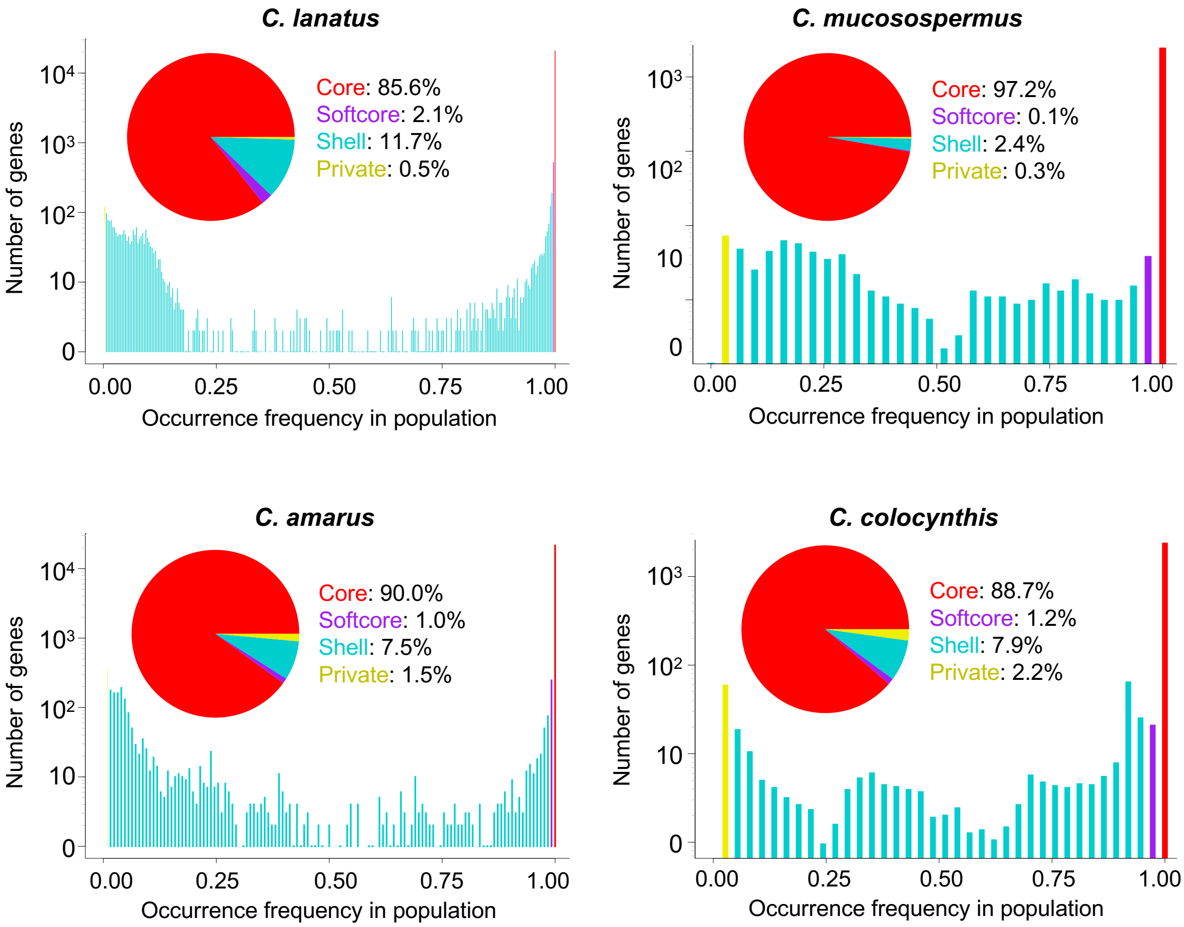

Figure S8 Compositions of the four species-specific pan-genomes. Core: present in all accessions; softcore: present in all but one; private: present in only one accession; shell: between softcore and private.

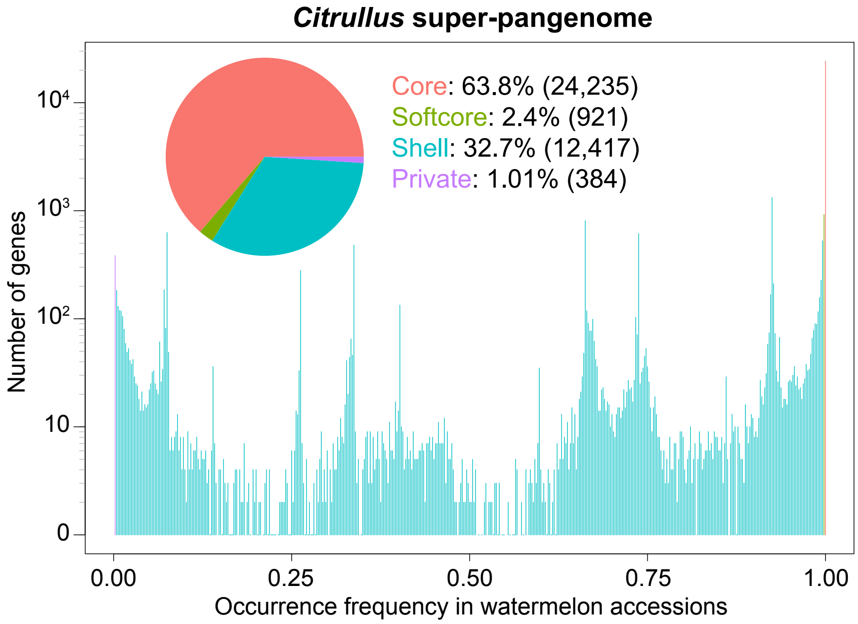

Figure S9 Compositions of the *Citrullus* super-pangenomes. Genes in the pan-genome were classified as core (present in all accessions), softcore (present in all but one), private (present in only one accession), and shell (between softcore and private).

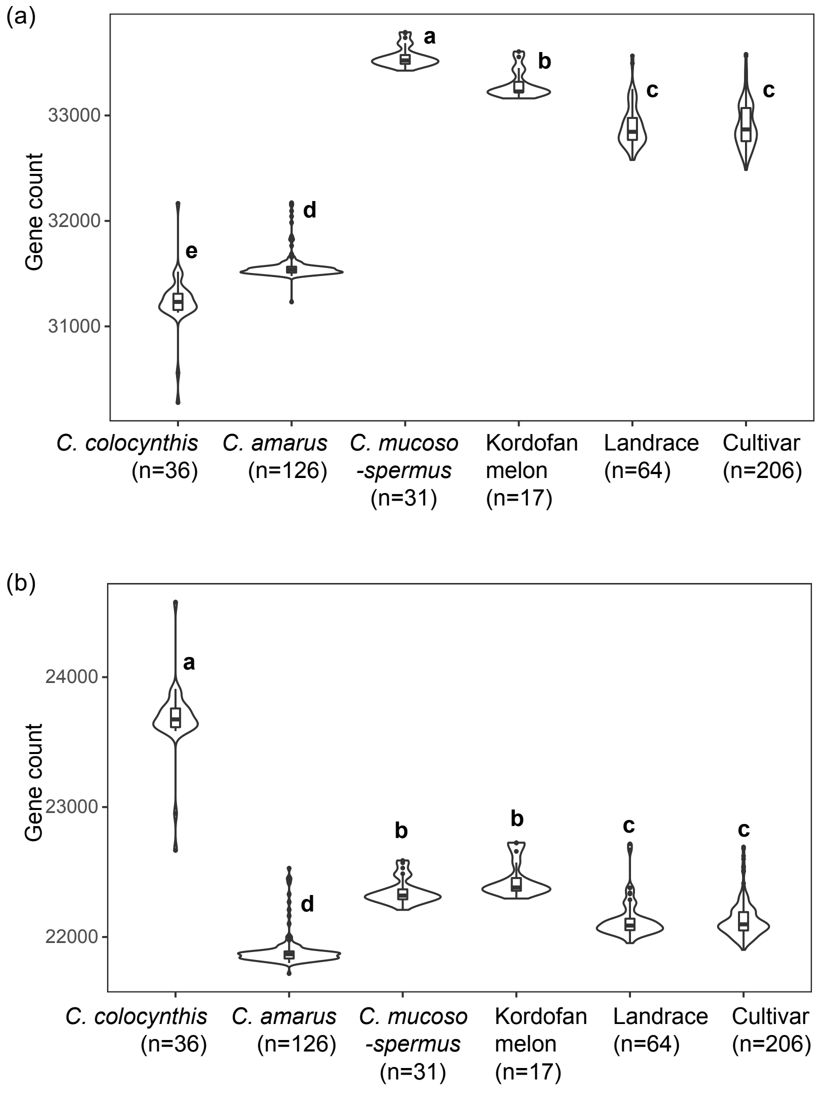

Figure S10 Numbers genes detected in individuals of different watermelon populations. (a) Distribution of numbers of loci, including orthologous loci without annotated genes. (b) Distribution of predicted genes only, excluding orthologous locations without predicted gene models. Different letters in the violin plots indicate the significant differences among different groups evaluated by Tukey's HSD test ($\alpha<0.05$).

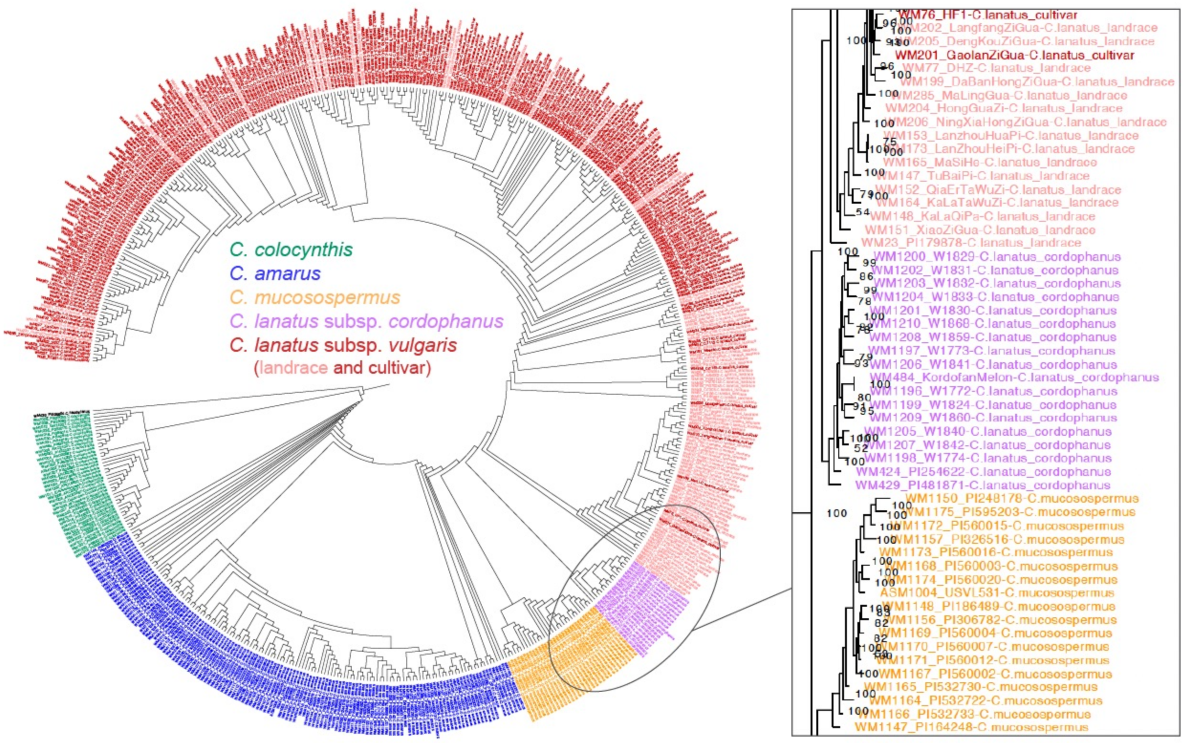

Figure S11 Maximum likelihood phylogenetic tree of wild and cultivated accessions.

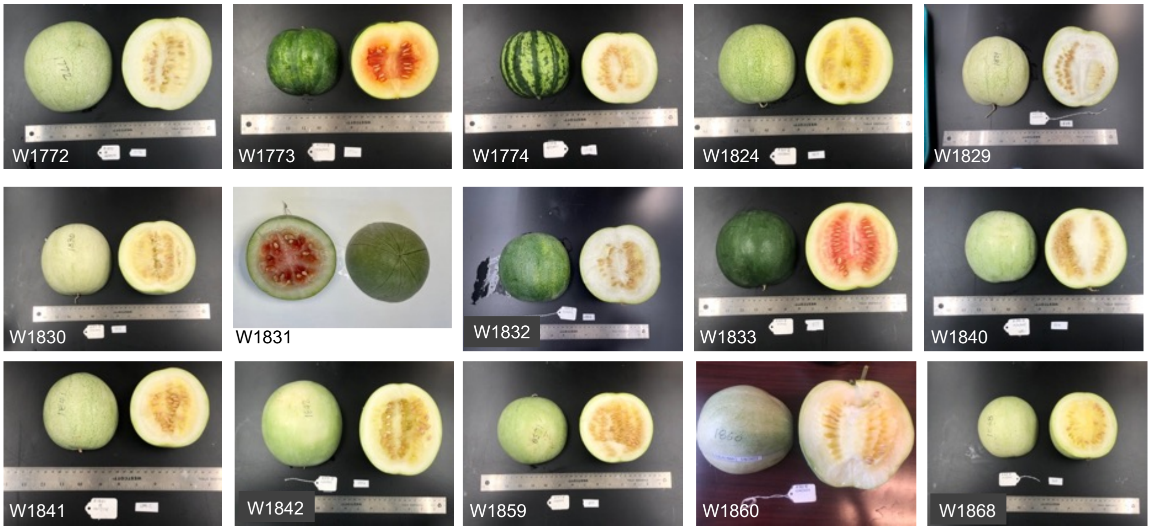

Figure S12 Mature fruits of 15 Kordofan melon accessions.

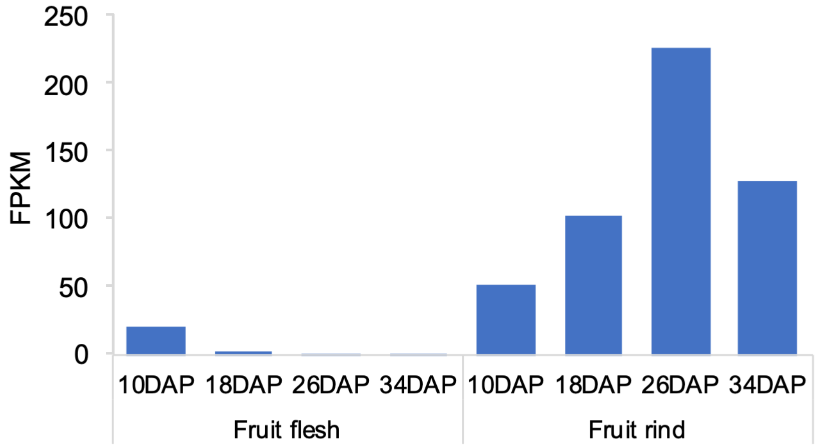

Figure S13 Expression levels of *Cla97C05G101010* in watermelon 97103 fruit flesh tissues at different developmental stages. DAP, days after pollination.

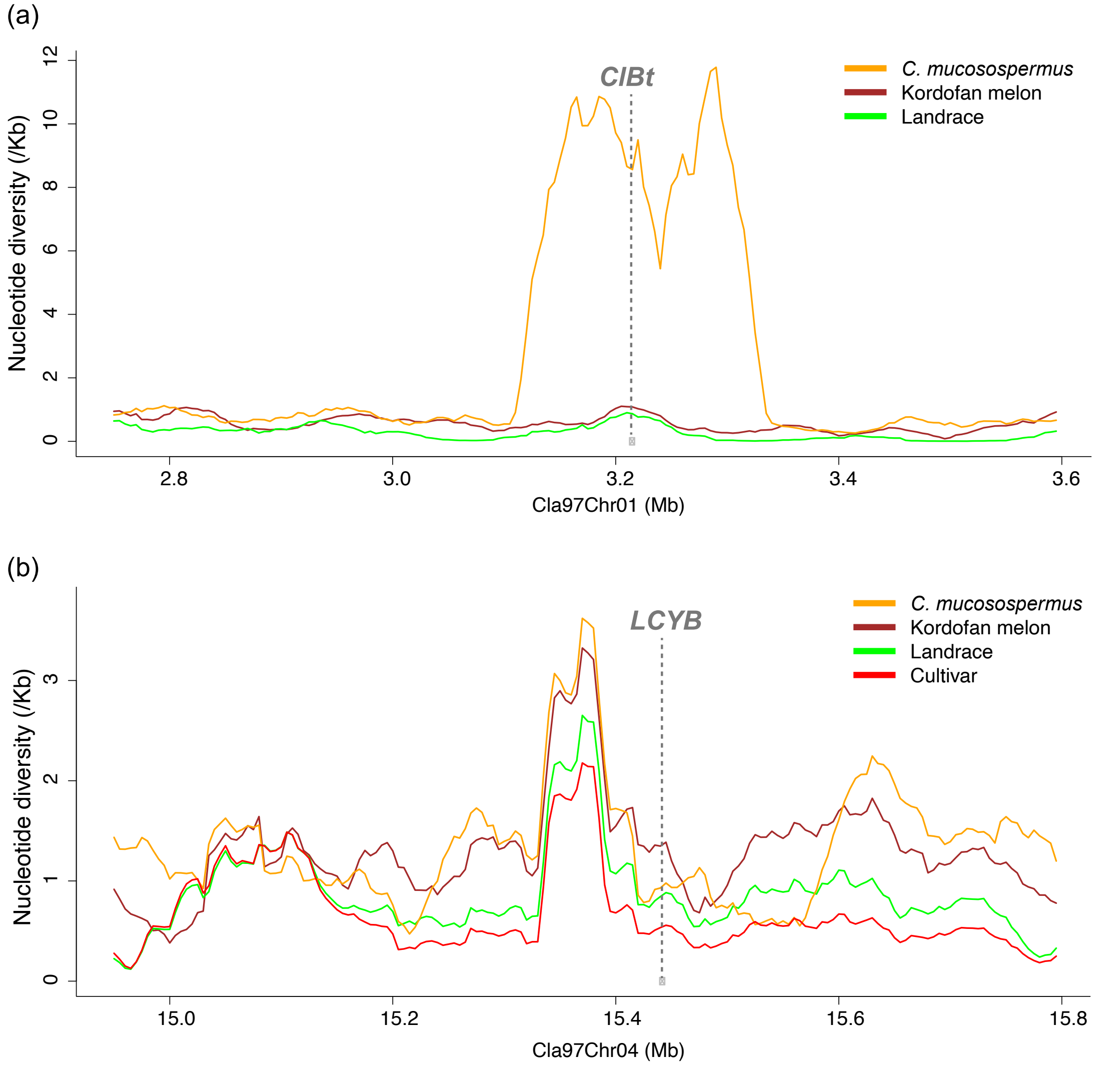

Figure S14 Nucleotide diversities in the genomic regions surrounding the *ClBt* (**a**) and *LCYB* (**b**) genes in different watermelon populations.

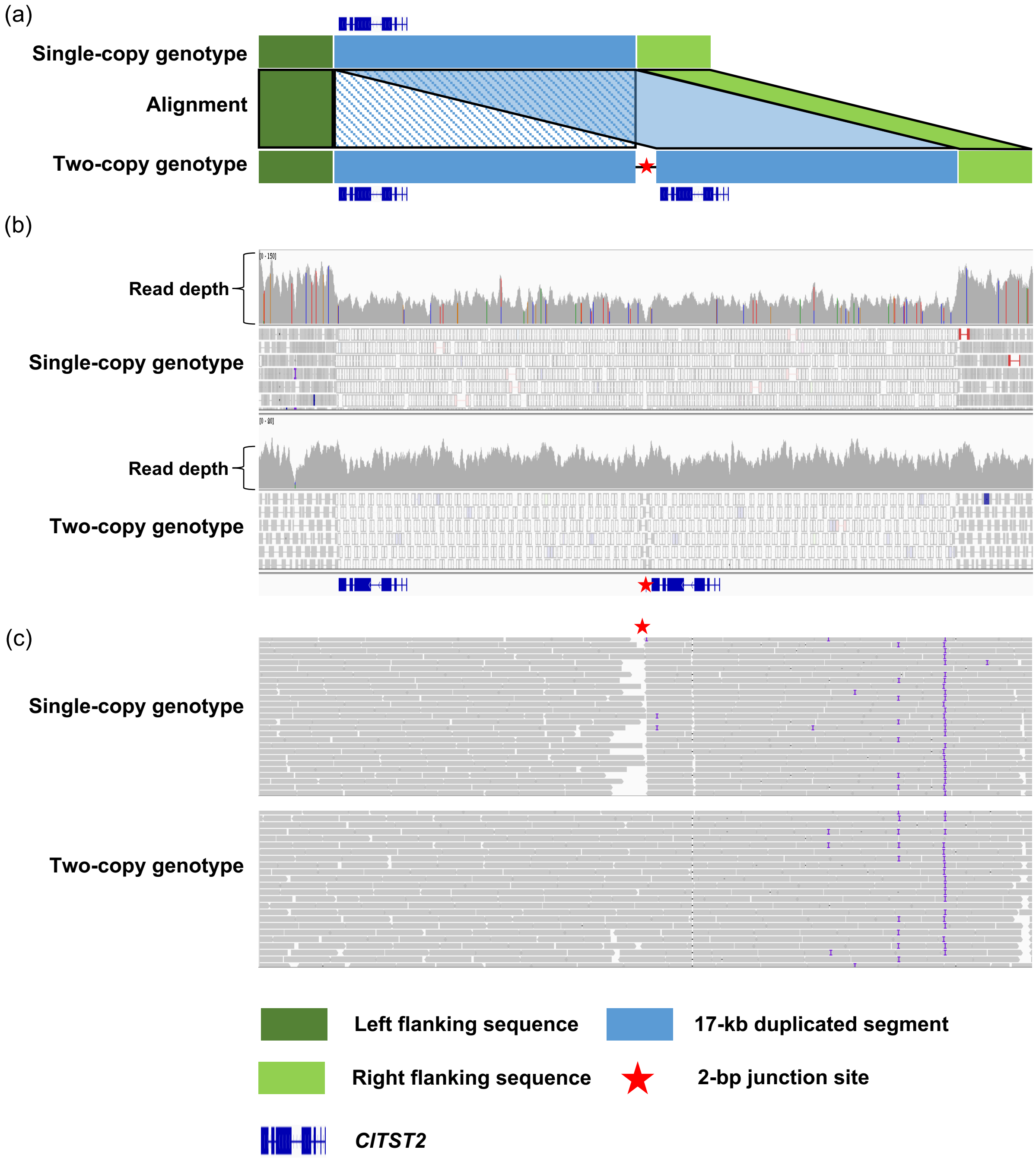

Figure S15 Read alignment at *ClTST2*. (a), diagram of the *ClTST2* tandem duplication. (b), read depth in the genomic region harboring the *ClTST2* tandem duplication. The accession with single-copy *ClTST2* has reduced read depth (about half) compared to the flanking regions (top panel), while the accession with two copies has similar length to the flanking regions (bottom panel). (c), read alignment at the 2-bp junction site unique to the *ClTST2* tandem duplication allele. Reads generated from the accessions with single-copy *ClTST2* cannot span the junction site (top panel), while those from the accession with two copies can (bottom panel).
